# Transcriptomic analysis identifies B-lymphocyte kinase as a therapeutic target for desmoplastic small round cell tumor cancer stem cell-like cells

**DOI:** 10.1038/s41389-023-00504-z

**Published:** 2024-01-04

**Authors:** Justin W. Magrath, Dane A. Flinchum, Alifiani B. Hartono, Shruthi Sanjitha Sampath, Tina M. O’Grady, Melody Baddoo, Liang Haoyang, Xiaojiang Xu, Erik K. Flemington, Sean B. Lee

**Affiliations:** 1 Department of Pathology and Laboratory Medicine, Tulane University School of Medicine, 1430 Tulane Ave, New Orleans, LA USA; 2 Present Address: Department of Molecular & Medical Pharmacology, University of California Los Angeles, 630 Charles E Young Dr. S., Los Angeles, CA 90095 USA

**Keywords:** Cancer stem cells, Sarcoma

## Abstract

Desmoplastic small round cell tumor (DSRCT) is an aggressive pediatric cancer caused by the EWSR1-WT1 fusion oncoprotein. The tumor is refractory to treatment with a 5-year survival rate of only 15–25%, necessitating the development of novel therapeutics, especially those able to target chemoresistant subpopulations. Novel in vitro cancer stem cell-like (CSC-like) culture conditions increase the expression of stemness markers (SOX2, NANOG) and reduce DSRCT cell line susceptibility to chemotherapy while maintaining the ability of DSRCT cells to form xenografts. To gain insights into this chemoresistant model, RNA-seq was performed to elucidate transcriptional alterations between DSRCT cells grown in CSC-like spheres and normal 2-dimensional adherent state. Commonly upregulated and downregulated genes were identified and utilized in pathway analysis revealing upregulation of pathways related to chromatin assembly and disassembly and downregulation of pathways including cell junction assembly and extracellular matrix organization. Alterations in chromatin assembly suggest a role for epigenetics in the DSRCT CSC-like state, which was further investigated with ATAC-seq, identifying over 10,000 differentially accessible peaks, including 4444 sphere accessible peaks and 6,120 adherent accessible peaks. Accessible regions were associated with higher gene expression, including increased accessibility of the CSC marker SOX2 in CSC-like culture conditions. These analyses were further utilized to identify potential CSC therapeutic targets, leading to the identification of B-lymphocyte kinase (BLK) as a CSC-enriched, EWSR1-WT1-regulated, druggable target. BLK inhibition and knockdown reduced CSC-like properties, including abrogation of tumorsphere formation and stemness marker expression. Importantly, BLK knockdown reduced DSRCT CSC-like cell chemoresistance, making its inhibition a promising target for future combination therapy.

## Introduction

Desmoplastic small round cell tumor (DSRCT) is an extremely deadly pediatric cancer with a 5-year survival rate of 15–25% [1–4]. DSRCT is caused by a chromosomal translocation that results in the creation of the *EWSR1-WT1* fusion oncogene, which dysregulates transcription and leads to tumor development [5, 6]. No targeted therapies have been established to-date and the current standard of care, multimodal therapy, including surgical resection, chemotherapy, and radiotherapy, is unable to adequately control the disease [2]. A cancer stem cell (CSC) subpopulation able to resist chemotherapy and initiate recurrence could explain the poor prognosis of DSRCT and is suggested by its high rate of recurrence and metastasis, resistance to chemotherapy, and expression of a diverse range of tissue-specific markers (neuron, muscle, epithelial, mesenchymal) [2, 3, 7]. This hypothesis is further bolstered by the high expression of *SOX2* in DSRCT relative to other fusion sarcomas and the finding that extraperitoneal metastatic DSRCT samples express higher levels of stemness markers (*SOX2* and *NANOG)* than intraperitoneal DSRCT tumors [8].

Recently, we utilized a novel culture condition to establish the first in vitro DSCT CSC model [8]. The DSRCT CSC model forms tumorspheres in vitro, has increased expression of stemness markers, and is able to resist doxorubicin treatment [8]. This model has the potential to provide important insights into the subpopulation of DSRCT, which is able to resist chemotherapy treatment and lead to tumor recurrence. Knockdown of *EWSR1-WT1* in our DSRCT CSC model reduced tumorsphere formation and *SOX2* expression, demonstrating the importance of the fusion oncoprotein to the DSRCT CSC state [8]. This observation aligns with recent work that found an enrichment of the *EWSR1-WT1* gene signature in recurrent/metastatic DSRCT when compared to primary tumors [9]. Together, these findings suggest that targeting the *EWSR1-WT1* fusion gene may be an effective strategy to eliminate both DSRCT bulk tumors and CSCs. However, to date no effective *EWSR1-WT1* targeting therapy has been established. An alternative strategy is to utilize a combination therapy where different components are able to target bulk tumors and CSCs.

In this study, we utilize our recently established DSRCT CSC model to gain insights into the DSRCT CSC-like population and identify therapies that can selectively target DSRCT CSC-like cells. RNA-seq analysis performed on three DSRCT cell lines grown in adherent or CSC-enriched sphere culture conditions enabled the identification of genes and pathways that are enriched in the DSRCT CSC model. Intriguingly, the altered genes and pathways between sphere and adherent culture conditions recapitulated many alterations in genes and pathways identified in the only published RNA-seq data from a pair of primary and recurrent DSRCT tumors. Our comprehensive transcriptomic analysis identified B-lymphocyte kinase (BLK) and lymphocyte-specific protein tyrosine kinase (LCK) as kinases that were upregulated in the DSRCT CSC model, controlled by the *EWSR1-WT1* fusion gene, and expressed more highly in recurrent versus primary DSRCT tumors. Drug inhibition and knockdown studies demonstrated the importance of BLK in the CSC state and identified BLK as a therapeutic target that can sensitize DSRCT CSC-like cells to doxorubicin chemotherapy.

## Materials/methods

### Cell lines and culture conditions

DSRCT cell lines (JN-DSRCT-1, BER-DSRCT, BOD-DSRCT, and SK-DSRCT2) have been previously described and validated to harbor the defining EWSR1-WT1 fusion [10–12]. Adherent culture: cells were cultured on tissue culture (treated) plates in DMEM/F12 media supplemented with 10% FBS (Gibco), 2 mM L-glutamine, 100 U/mL penicillin, and 100 μg/mL streptomycin (ThermoFisher, Waltham, MA). Sphere culture: as previously described cells were seeded on non-treated plates (Costar® 6-well Clear Not Treated Multiple, Corning) in a 1:1 mixture of DMEM/F12 and Neurobasal Media supplemented with 2 mM L-glutamine, 100 U/mL penicillin, and 100 μg/mL streptomycin (ThermoFisher, Waltham, MA).

### Light microscopy

Light microscopy was performed with Nikon Eclipse 80i microscope using NIS-Elements software for image capture. Images of DSRCT cells in adherent culture were taken near confluence and in sphere conditions were taken at 4 days after induction of sphere formation. Images for both conditions were taken at ×10 magnification.

### RNA sequencing and analysis

RNAs were isolated from cells in the sphere or adherent culture for 4 days. RNAs were prepared with RNeasy Plus kit (Qiagen, Hilden, Germany). Sequencing libraries were created from 500 ng of total RNA using the Illumina TruSeq Stranded Total RNA kit with Ribo-Zero (San Diego, CA) following the manufacturer’s instruction. Library fragment size was verified using the Agilent 2100 Bioanalyzer (Agilent, Santa Clara, CA), and the concentrations were determined using Qubit (ThermoFisher). Illumina Novaseq 6000 (San Diego, CA) was utilized for 75 bp paired-end read sequencing. Gene counts were generated by alignment to human transcriptome GRCh38 using STAR in RSEM [13]. Differential gene expression analysis was performed using DESeq2 [14]. Overrepresentation Analysis and Gene Set Enrichment Analysis were performed using the clusterProfiler package in Bioconductor [15, 16]. Publicly available RNA-seq data of EWSR1-WT1 knockdown in JN-DSRCT-1 and BER-DSRCT cell lines (GSE137561) and primary versus recurrent DSRCT tumors (GSE230603) were obtained from GEO.

### ATAC and ChIP sequencing and analysis

ATAC-seq was performed as previously described on BER-DSRCT cells cultured for 4 days in the sphere or adherent conditions using 50,000 cells [17]. ATAC-seq libraries were sequenced to 50 million reads on NovaSeq 6000 (Illumina) with 150 bp paired-end sequencing. Quality control was performed with FASTQC and deduplication was performed with bbmap-clumpify. Bowtie2 [18, 19] was used to align sequences to hg38 followed by narrow peak calling with MACS2 [20]. Differential accessibility was performed using the DiffBind R package [21] and annotations were established with ChIPseeker [22]. HOMER was used for motif enrichment analysis [23]. The Integrative Genomics Viewer (IGV) was used for visualization of peaks [24]. BigWig and peak files of previously published WT1 ChIP-seq in JN-DSRCT-1 cells were obtained from GEO (GSE156277) and visualized with IGV.

### Single-cell RNA sequencing and analysis

DSRCT cells were grown in sphere or adherent culture for 7 days and harvested into single cells with trypsin. Cell numbers and viabilities were validated by Cellometer Automated Cell Counter (Nexcelom Bioscience, Lawrence, MA, USA) prior to single-cell RNA-seq library preparation.

For feature barcode technology and 10x single-cell RNA-seq assay, live cells per sample were targeted using feature barcode oligonucleotide conjugated to a lipid. Labeled cells from each sample were pooled, and 10x Single Cell 3’ RNA-seq technology was applied to generate both cell multiplexing libraries and cDNA libraries. Briefly, 2000 viable single cells from each sample were labeled with unique cell multiplexing oligo (CMO) provided by 10x Genomics (10X Genomics Inc, CA). Labeled cells from each sample were pooled and partitioned into nanoliter-scale Gel Beads-In-EMulsion (GEMs). Feature barcode molecules were captured directly by oligonucleotides presented on the Gel Beads inside a GEM during GEM-RT. Cell multiplexing libraries comprising dual index NN set A (10X Genomics Inc, CA) were generated by index PCR amplification. Meanwhile, Full-length barcoded cDNAs were also generated and amplified by PCR to obtain sufficient mass for library construction. Following enzymatic fragmentation, end-repair, A-tailing, and adaptor ligation, single cell 3’ libraries comprising dual index TT set A (10X Genomics Inc, CA) were generated. Library quality controls were performed by using Agilent High Sensitive DNA kit with Agilent 2100 Bioanalyzer (Agilent Technologies, Palo Alto, CA, USA) and quantified by Qubit 2.0 fluorometer (ThermoFisher Scientific). Pooled libraries at a final concentration of 750 pM were sequenced with paired-end dual index configuration by Illumina NextSeq 2000. ScRNA-seq analysis utilized CellRanger and Seurat [25], as detailed in the supplementary methods.

### RNA isolation and real-time qPCR analysis

Total RNA was isolated with RNA-STAT60 (Tel-Test, Friendswood, TX). iScript cDNA Synthesis Kit (Bio-Rad, Hercules, CA) was used to reverse transcribe 500 ng of RNA to generate cDNA. Relative transcript levels were analyzed by real-time qPCR using SYBR Green (SsoAdvanced Universal SYBR Green Supermix, Bio-Rad) and calculated by the comparative Ct method normalized against human ACTB (β-ACTIN). Primers are listed in Supplementary Table 1.

### Protein isolation and western blot analysis

Cell lysates were prepared in RIPA lysis buffer supplemented with complete EDTA-Free Protease Inhibitor Cocktail (Sigma-Aldrich) and phosphatase inhibitors: 1 mM NaF and 2 mM Na_3_VO_4_. Proteins were resolved in 10% SDS-PAGE gels and transferred onto a 0.45 µm nitrocellulose membrane (Bio-Rad). Membranes were blocked with 5% non-fat milk and incubated with primary antibodies at 4^o^C overnight, followed by room temperature incubation of secondary antibodies LI-COR IRDye 800CW goat anti-Mouse (#926-32210, 1:15,000 dilution) or LI-COR IRDye 680RD goat anti-Rabbit (#926- 68071, 1:15,000 dilution). Images were taken on LI-COR Odyssey CLx (Lincoln, NE). At least two independent immunoblots were performed for each experiment, with a representative immunoblot shown. Antibodies are listed in Supplementary Table 2.

### Cell viability and chemoresistance assays

The CCK-8 assay (Sigma-Aldrich) was utilized to assess cell viability and chemoresistance per the manufacturer’s directions. For cell viability assays, cells were seeded in sphere media in non-treated 96-well plates at day 0. After 24 h of culture, media with either vehicle control or 1 or 10 μM of dasatinib or PP2 were added to cells. CCK-8 was performed 3, 5, and 7 days after inhibitor treatment (*n* = 4). For chemoresistance assays, cells with doxycycline (dox)-inducible shRNA against *BLK* were seeded in sphere culture media with (1μg/mL) or without dox in non-treated 96-well plates for 4 days respectively before doxorubicin addition. On day 4, media with 10 nM to 10 μM of doxorubicin (Millipore Sigma) with or without dox were added to cells. At 72 hours, CCK-8 assay was performed to assess viability. For all experiments, absorbance was measured using Clariostar microplate reader (BMG Labtech, Cary, NC).

### Generation of dox-inducible shRNA cell lines

Dox-inducible LT3-GEPIR vector [26] was used to generate stable cell lines. BER-DSRCT and SK-DSRCT2 cell lines with dox-inducible shRNA against the *WT1* 3′UTR (5′ GCAGCTAACAATGTCTGGTTA 3′) have been previously established^8^. JN-DSRCT-1 and BER-DSRCT cell lines with shRNA knocking down *BLK* (5’ GAGCTGATCAAGCACTATAAG 3’) and *LCK* (5’ TCACATGGCCTATGCACATAT 3’) were established by inserting annealed oligonucleotides into XhoI and EcoRI sites of the LT3-GEPIR vector. Lentivirus was generated by co-transfecting HEK293T cells with the LT3-GEPIR-shRNA lentiviral vector and ViraPower lentiviral packaging mix (Invitrogen) using Lipofectamine3000 (Thermofisher Scientific). Viral supernatants were collected 48-, 72-, and 96-hours post-transfection and concentrated with LentiX-Concentrator (Takara Bio, San Jose, CA). JN-DSRCT-1 and BER-DSRCT cells were transduced with LT3-GEPIR virus in the presence of polybrene (10 µg/ml) for 16 hours. Cells were selected with puromycin (0.5 µg/mL) 48 h post-transduction. Stable cell lines were validated by RT-qPCR and Western blot analyses with or without dox.

### ChIP-qPCR

ChIP assay was adapted and performed as previously described [27] using anti-H3K27ac (Cell Signaling) or rabbit IgG (Cell Signaling) antibodies. Cells were crosslinked with formaldehyde for 5 minutes, neutralized with glycine, washed twice, and lysed in Farnham Buffer. Chromatin was fragmented by sonication with Covaris M220 Focused-ultrasonicator. Samples were incubated with antibodies overnight at 4 C, pulled down with Protein G dynabeads (Invitrogen), washed, eluted, and purified with DNA spin columns (Epoch Life Science). qPCR was performed using SYBR Green (SsoAdvanced Universal SYBR Green Supermix, Bio-Rad) with ChIP-qPCR primers in Supplementary Table 3.

## Results

### Transcriptomic analysis shows downregulation of growth and upregulation of chromatin assembly in DSRCT CSC-like cells

**Fig. 1:**
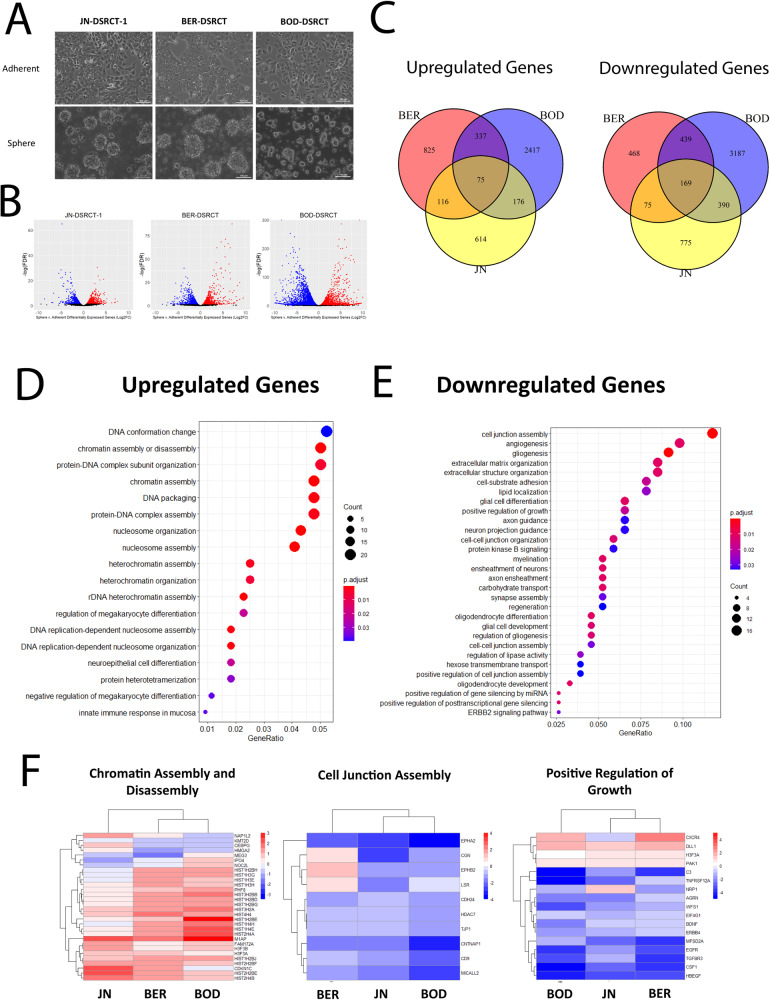
DSRCT CSC gene expression. **A** Light microscopy of three DSRCT cell lines grown in adherent or sphere culture conditions (scale bar = 100 µm). **B** Volcano plots of differentially expressed genes in sphere versus adherent culture (red=significantly upregulated in spheres, blue=significantly upregulated in adherent culture, FDR < 0.05). **C** Venn diagram of commonly upregulated and downregulated genes between three DSRCT cell lines in sphere versus adherent culture (log2FC > |0.58|, FDR < 0.05). **D**, **E** Overrepresentation of sphere-upregulated genes (**D**) and downregulated genes (**E**) in GO-BP pathways. **F** Heatmaps of differentially expressed genes in chromatin assembly and disassembly, cell junction assembly, and positive growth regulation GO-BP pathways (log2FC sphere/adherent gene expression).

To gain insight into the differences in gene expression between normal DSRCT culture conditions (adherent culture) and our novel DSRCT CSC model (sphere culture), RNA sequencing (RNA-seq) was performed on three DSRCT cell lines (JN-DSRCT-1, BER-DSRCT, BOD-DSRCT) cultured in adherent or sphere culture for 4 days (Fig. 1A). Consistent with our previous work, we found upregulation of stemness markers *SOX2* and *NANOG* in the DSRCT sphere culture condition (Supplementary Fig. 1A). Using a log2fold-change threshold of >|0.58| and adjusted *p* value threshold of <0.05, we identified 981 upregulated and 1409 downregulated genes in JN-DSRCT-1 sphere versus adherent culture, 1356 upregulated and 1152 downregulated genes in BER-DSRCT sphere versus adherent culture, and 3027 upregulated and 4187 downregulated genes in BOD-DSRCT sphere versus adherent culture (Fig. 1B). Comparing the differentially expressed genes between cell lines, we identified 75 genes upregulated under CSC-enriched conditions in all three cell lines and 169 downregulated genes under CSC-enriched conditions in all three cell lines (Fig. 1C).

Overrepresentation analysis on the 169 commonly downregulated genes identified a variety of gene ontology (GO) biological processes with statistically significant overrepresentation, including cell junction assembly, extracellular matrix organization, and positive regulation of cell growth (Fig. 1E, Supplementary Data 1). This pathway enrichment was further validated by gene set enrichment analysis (GSEA) (Supplementary Fig. 1B). Heat maps of the significantly altered genes in the cell junction assembly gene set and positive regulation of the growth gene set to support this finding, with most of the significantly altered genes in both these gene sets showing downregulation (Fig. 1F). An overrepresentation of downregulated genes in the pathway for positive regulation of growth is consistent with our previous work showing DSRCT CSCs have a more quiescent state with a lower rate of proliferation and lower percentage of cells in mitosis [8]. This analysis suggests that the downregulation of genes, including EGFR and ERBB4 may help explain the quiescent state of DSRCT CSCs.

Overrepresentation analysis on the 75 commonly upregulated genes did not identify any overrepresented GO biological processes. Broadening the list to include genes upregulated in sphere versus adherent culture in at least two of our three DSRCT cell lines led to the identification of pathways with statistically significant overrepresentation (Fig. 1C, Supplementary Data 1). The overrepresented pathways with the highest statistical significance were related to chromatin alterations, including chromatin assembly and disassembly, protein-DNA complex subunit organization, and nucleosome organization. GSEA similarly demonstrated enrichment of chromatin assembly and disassembly (Supplementary Fig. 1B). This finding suggests epigenetic alterations may play a role in the DSRCT CSC state. Upregulated genes related to chromatin assembly and disassembly included histones (*HIST2H4b*, *HIST2H2BE*, *HIST1H2BJ*), the histone-modifying enzyme *PHF8*, and the protein *FAM172A* which has roles in neural crest development and heterochromatin assembly involving small nuclear RNAs [28] (Fig. 1F).

Recently, we demonstrated that despite increased resistance to chemotherapy drugs, including doxorubicin, the DSRCT CSC model remains sensitive to knockdown of *EWSR1-WT1* [8]. To understand the role of the fusion protein in the DSRCT CSC model, we examined the expression of *EWSR1-WT1*-regulated genes in sphere versus adherent culture. A heatmap of the Gedminas et al. EWSR1-WT1 regulated signature showed that genes downregulated by *EWSR1-WT1* are further downregulated in sphere culture, consistent with our previous finding that the fusion is more highly expressed in spheres compared to adherent cells (Supplementary Fig. 2A) [8]. However, the heat map did not show a further induction of *EWSR1-WT1* upregulated genes in sphere versus adherent culture. Both these trends were confirmed by GSEA, which showed significant downregulation of *EWSR1-WT1* downregulated genes in all three cell lines but no significant differences in *EWSR1-WT1* upregulated genes (Supplementary Fig. 2B). The selective alteration of EWSR1-WT1 downregulated but not upregulated genes is an unexpected finding that may be influenced by epigenetic changes or alterations in the transcriptional machinery between adherent and sphere cells and warrants future investigation.

### DSRCT CSC model recapitulates gene expression observed in recurrent versus primary DSRCT tumor pair

**Fig. 2:**
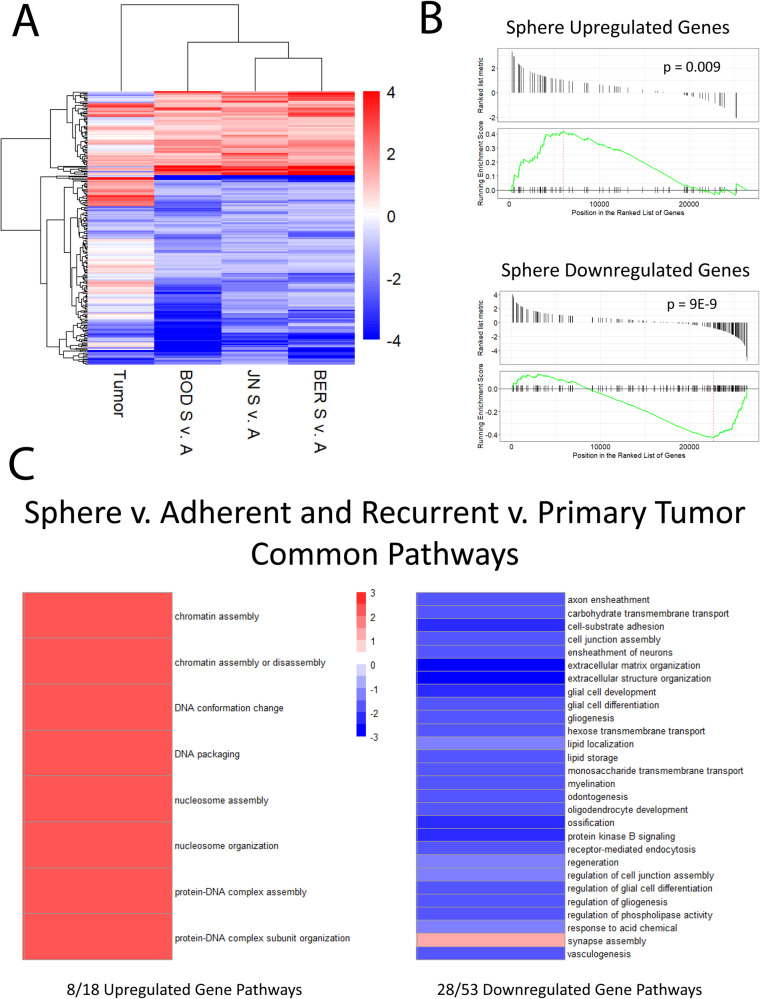
DSRCT CSC model recapitulates expression of recurrent versus primary tumor. **A** Heatmap of common sphere-upregulated and downregulated genes. Log2FC gene expression is displayed for the recurrent/primary DSRCT tumor pair and sphere (S)/adherent (A) cell lines. **B** GSEA of sphere-upregulated and downregulated gene sets on recurrent/primary tumor gene expression data. **C** Heatmap of normalized enrichment scores for pathways that are overrepresented in sphere versus adherent culture and enriched in recurrent versus primary DSRCT tumors (red = enriched in recurrent tumor, blue = enriched in primary tumor).

To evaluate the clinical relevance of the genes and pathways differentially expressed in the sphere versus adherent culture conditions, these differentially expressed genes and pathways were compared to the altered genes and pathways in the only published RNA-seq data from a pair of primary and recurrent DSRCT tumors harvested from the same patient (GSE230603). The primary sample was harvested from the retroperitoneal pelvic cavity of a 24-year-old male shortly after diagnosis. The recurrent/metastatic tumor was harvested from the same patient 5 years later from the parenchyma of the liver, making the second tumor both recurrent and metastatic. Since CSCs are critical to recurrence and metastasis, we hypothesized that genes and pathways upregulated in sphere versus adherent culture would be upregulated in recurrent versus primary DSRCT tumors, and likewise, downregulated genes and pathways in sphere versus adherent culture would be downregulated in recurrent versus primary DSRCT tumors. A heat map examining the commonly upregulated and downregulated genes in sphere versus adherent culture in all three cell lines showed similar gene expression alterations in the recurrent versus primary tumor samples (Fig. 2A). GSEA on the recurrent versus primary tumor RNA-seq data using gene sets consisting of the 75 commonly upregulated genes and 177 commonly downregulated genes in sphere versus adherent culture further confirmed this observation as the sphere-upregulated gene set showed upregulation (*p* = 9E-3) and the sphere-downregulated gene set showed downregulation in the recurrent versus primary DSRCT tumor pair (*p* = 9E-9) (Fig. 2B).

Examining GO biological processes overrepresented in sphere versus adherent culture in the recurrent versus primary tumor data set, we found that 8/18 upregulated pathways and 28/53 downregulated pathways in spheres showed statically significant enrichment in the recurrent versus primary tumor samples. All except one of the pathways found to be overrepresented in sphere versus adherent cells showed the same directionality in recurrent versus primary tumors. Commonly upregulated pathways included chromatin assembly, DNA conformation change, and nucleosome assembly, while commonly downregulated pathways included cell junction assembly, glial cell development, and protein kinase B signaling (Fig. 2C). The lone pathway with different directionality between sphere versus adherent cells and recurrent versus primary tumors was synapse assembly which was overrepresented in downregulated genes in sphere versus adherent cells but was upregulated in recurrent versus primary tumors. While this suggests the CSC model does not completely mimic the metastatic environment, the high concordance in gene expression alterations between sphere versus adherent cells and recurrent versus primary tumors suggests that the DSRCT CSC model recapitulates many clinically relevant gene and pathway alterations and could be valuable for identifying targets involved in DSRCT recurrence.

### Single-cell sequencing reveals sphere-enriched subpopulations

**Fig. 3:**
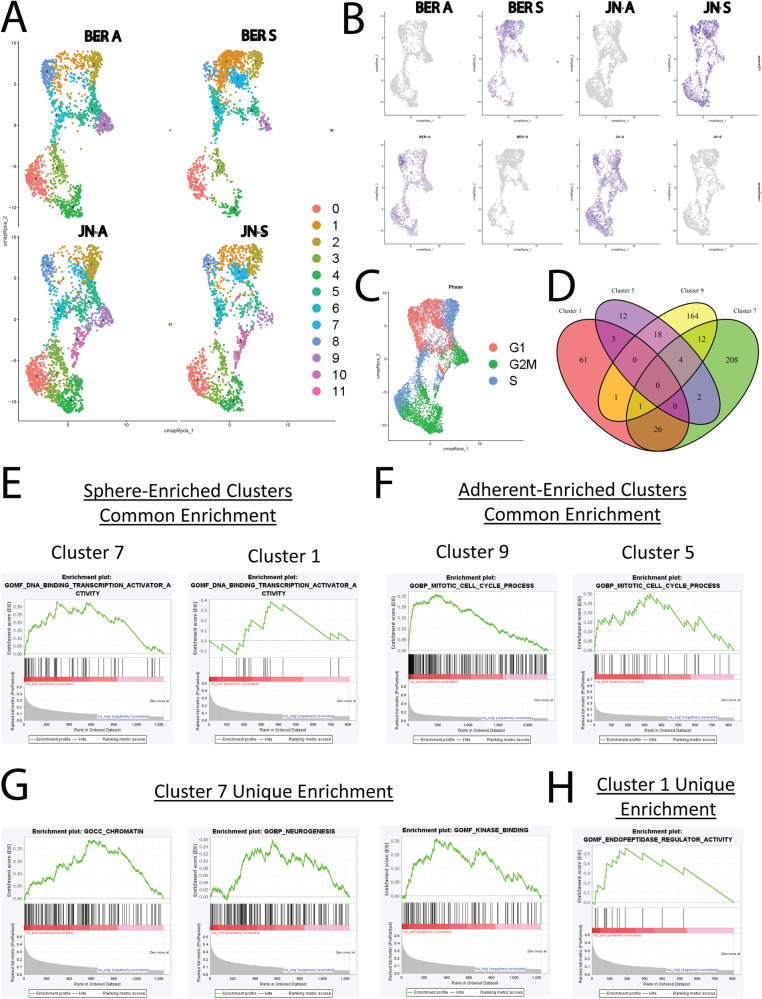
Identification of CSC-enriched subpopulations. **A** UMAP projection of BER-DSRCT and JN-DSRCT-1 cells in adherent (A) or sphere (S) culture divided into 11 clusters. **B** Expression of the sphere-enriched signature (top row) and adherent-enriched signature (bottom row) in single cells. **C** UMAP projection of DSRCT single cells labeled by cell cycle phase. **D** Venn diagram of enriched pathways in clusters 1, 7, 5, and 9. **E** GSEA of DNA Binding Transcription Activator Activity gene set on clusters 1 and 7. **F** GSEA of Mitotic Cell Cycle Process gene set on clusters 5 and 9. **G** GSEA of Chromatin, Neurogenesis, and Kinase Binding gene sets on cluster 7. **H** GSEA of Endopeptidase Regulatory Activity gene set on cluster 1.

Single-cell RNA sequencing (scRNA-seq) was performed on JN and BER-DSRCT cells to examine transcriptional heterogeneity within and between DSRCT sphere and adherent culture conditions. A total of 7996 cells were sequenced with a median gene count of 5163 genes per cell. Each cell condition was composed of cells with a variety of transcriptional signatures that clustered into eleven groups using RPCA (Fig. 3A, Supplementary Fig. 3A). Cluster 10 was only found in JN-DSRCT-1 cells, suggesting a cell line-specific transcriptomic state, while all other clusters contained cells from each of the four conditions (JN A, JN S, BER A, BER S) (Fig. 3A). Two clusters (1 and 7) were enriched in spheres versus adherent cells for both JN- and BER-DSRCT cell lines (Fig. 3A). Conversely, clusters 5 and 9 were depleted in spheres. While a variety of clusters demonstrated SOX2 expression, this expression was most robust in the sphere-enriched clusters 1 and 7 (Supplementary Fig. 3B). Pathway analysis on our bulk RNA-seq derived sphere-upregulated and downregulated gene sets showed enrichment of sphere-upregulated genes in sphere culture scRNA-seq data and enrichment of sphere-downregulated genes in adherent culture scRNA-seq data, recapitulating our previous bulk RNA-seq findings (Fig. 3B). While the sphere-upregulated gene signature was identified in spheres from across the clusters, most prominent expression was found in the sphere-enriched clusters 1 and 7 (Fig. 3B). Analysis of cell cycle gene signatures found clusters 1 and 7 contain cells predominantly in G1/G0 phase (Fig. 3C), consistent with our previous findings suggesting spheres are more quiescent. To better understand the similarities and differences in the two sphere-enriched and two adherent-enriched clusters, we performed GSEA on each cluster versus all others and identified 94, 253, 41, and 200 enriched Gene Ontology pathways in Clusters 1, 7, 5, and 9, respectively. Twenty-seven enriched pathways were in common between sphere-enriched clusters 1 and 7, including DNA Binding Transcription Activator Activity, Negative Regulation of Cell Differentiation, and Cell Motility (Fig. 3D, E, Supplementary Data 2). Twenty-two pathways were commonly enriched in adherent-enriched clusters 5 and 9 (Fig. 3D) and were nearly all related to cell division, including Mitotic Cell Cycle Process, Cell Division, and Organelle Fission (Fig. 3F, Supplementary Data 2). The differences in pathway enrichment between the two sphere-enriched clusters suggest the existence of two distinct DSRCT CSC-like populations. The Cluster 7 CSC-like subpopulation had an enrichment of Chromatin and Chromatin Binding in line with the enrichment of a plethora of chromatin assembly-related pathways in the sphere culture bulk RNA-seq data. Cluster 7 also showed enrichment of DNA Repair, Neurogenesis, Regulation of Axonogenesis, and Kinase Binding (Fig. 3G). In contrast, Cluster 1 was uniquely enriched in pathways related to protein catabolism, including Endopeptidase Regulatory Activity and Positive Regulation of Proteolysis (Fig. 3H). Overall, the scRNA-seq analysis not only validated our previous bulk RNA-seq findings but also identified DSRCT subpopulations that undergo dynamic changes between adherent and sphere culture conditions. Analysis of these specific subpopulations enabled the identification of novel enriched pathways, which may contribute to observed phenotypic differences between sphere and adherent cell states.

### Chromatin accessibility alterations are associated with gene expression changes

**Fig. 4:**
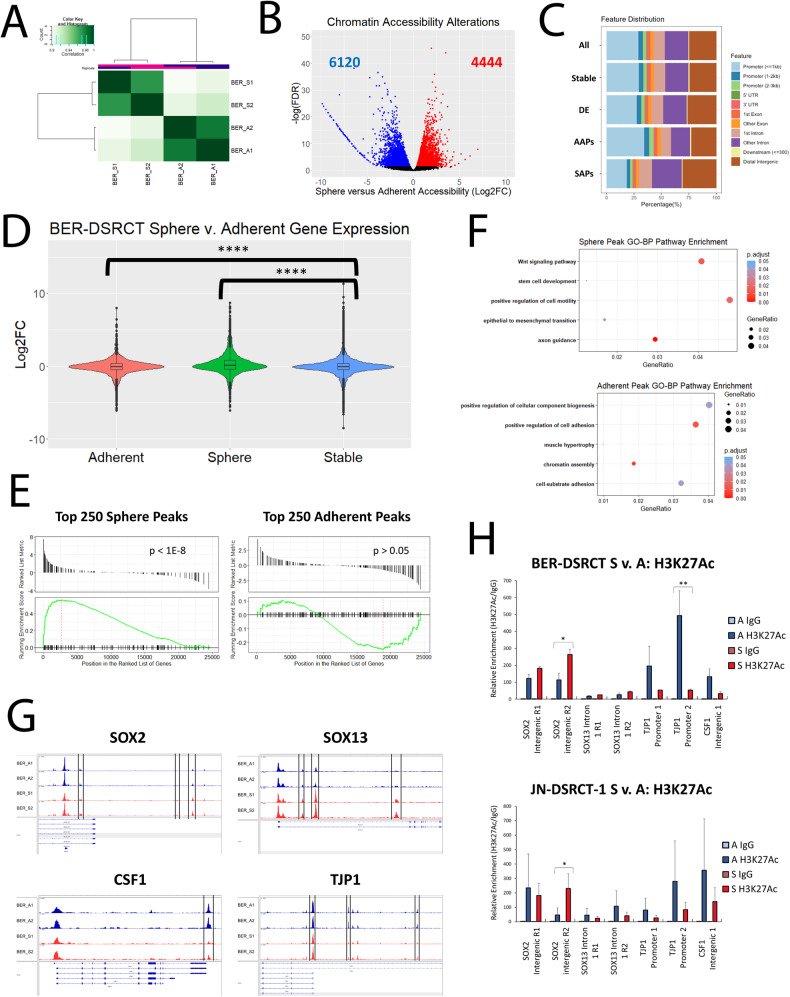
DSRCT CSC chromatin accessibility alterations. **A** Pearson correlation between ATAC-seq biological replicates (S = sphere, A = adherent). **B** Volcano plot of differentially accessible chromatin regions in sphere versus adherent BER-DSRCT cells (red = sphere-enriched peaks, blue = adherent-enriched peaks). **C** Feature distribution of ATAC-seq peaks (all = all peaks, stable = peaks with FDR > 0.05, DE = peaks with FDR < 0.05, AAPs = peaks with FDR < 0.05 & sphere/adherent fold < 0, SAPs = peaks with FDR < 0.05 & sphere/adherent fold > 0). **D** Log2FC (sphere/adherent) RNA expression of closest gene to each ATAC-seq peak for adherent peaks (AAPs), sphere peaks (SAPs), and stable peaks (FDR > 0.05) (****p < 0.0001, student *t* test). **E** GSEA of BER-DSRCT sphere/adherent RNA expression data on the sets of the top 250 SAPs and top 250 AAPs. **F** GO-BP pathways enriched for SAPs and AAPs. **G** BER-DSRCT adherent (blue) and sphere (red) ATAC-seq tracks for *SOX2*, *SOX13*, *CSF1*, and *TJP1*. **H** ChIP-qPCR of differentially accessible regions in DSRCT cells grown in adherent (A) or sphere (S) culture and immunoprecipitated with anti-H3K27ac antibody or control IgG (*n* = 3, *p < 0.05, **p < 0.01).

Having found enrichment in pathways related to chromatin assembly in both bulk RNA-seq analysis and sphere-enriched Cluster 7, we hypothesized that chromatin alterations may play a role in the gene expression changes between adherent cells and spheres. To understand this potential relationship, we performed ATAC-seq on BER-DSRCT cells in the sphere and adherent culture conditions to identify alterations in chromatin accessibility [17]. We found a high correlation between ATAC-seq signal in each set of biological replicates with *r* = 0.97 for sphere culture replicates and *r* = 0.98 for adherent culture replicates (Fig. 4A). Using DiffBind [21], we identified over 62,000 peaks of which 4444 peaks were significantly more accessible in spheres (SAPs: sphere accessible peaks) and 6120 peaks were significantly more accessible in adherent cells (AAPs: adherent accessible peaks) (Fig. 4B). Compared to the set of all peaks, SAPs were more commonly located in intergenic or intronic regions, while AAPs were more commonly located in the promoter region (Fig. 4C). Motif analysis with HOMER identified motifs enriched in SAPs and AAPs. The top motifs in SAPs were HEB, E2A, and LEF1 (Supplementary Fig. 4A), while the top motifs in AAPs were CArG, Fra1, and BATF (Supplementary Fig. 4B). Consistent with their higher expression in spheres, SOX2 and NANOG motifs were enriched among SAPs but not AAPs. The transcription factor ASCL1, previously found to be important for DSRCT growth or survival [29], was also enriched in SAPs but not AAPs. Intriguingly, the WT1 motif was enriched among both SAPs and AAPs, potentially suggesting that EWSR1-WT1 plays a role in either chromatin accessibility alterations or downstream gene expression changes.

The relationship between chromatin accessibility and gene expression was next explored by comparing the average change in gene expression for genes associated with SAPs, AAPs, and stable peaks (Fig. 4D). Genes associated with SAPs were more highly expressed in spheres than adherent cells with an average log2FC (sphere/adherent) of 0.34, while genes associated with AAPs were more highly expressed in adherent culture with an average log2FC of −0.12 (Fig. 4D). Both these changes were significant in comparison to the gene expression of stable peaks which had an average RNA expression log2FC of −0.05. As a second metric to assess the relationship between chromatin accessibility and gene expression, we performed GSEA on BER sphere versus adherent RNA-seq data (Fig. 1B) using gene sets consisting of genes associated with the top 250 SAPs or AAPs (Fig. 4E). There was a significant positive enrichment of the top 250 SAPs (*p* < 10^-8^), while there was a trend toward negative enrichment for the top 250 AAPs which was not statistically significant. Overrepresentation analysis identified pathways enriched among SAPs and AAPs (Fig. 4F). Enriched SAP pathways included Wnt signaling, positive regulation of motility, and epithelial to mesenchymal transition, while enriched AAP pathways included positive regulation of cell adhesion and positive regulation of cellular component biogenesis. Specific genes with altered chromatin accessibility and RNA expression include *SOX2*, *SOX13*, *CSF1*, and *TJP1* (Fig. 4G). *SOX2* and *SOX13* both have increased accessibility and increased RNA expression in the sphere state. *SOX2* tracks were remarkable for three significantly increased accessibility peaks in the intergenic region, while *SOX13* tracks were remarkable for three significantly increased accessibility peaks in intron 1. Conversely, *CSF1* and *TJP1* are involved in cell adhesion and were found to have decreased accessibility and decreased RNA expression in sphere culture. *CSF1* tracks showed one significantly reduced accessibility peak in the intergenic region. *TJP1* tracks demonstrated three significantly reduced accessibility peaks in the promoter region. To gain better insight into the epigenetic alterations responsible for these DNA accessibility changes, we performed Chromatin immunoprecipitation (ChIP) with an anti-H3K27 acetylation (ac) antibody followed by qPCR (Fig. 4H). We found greater enrichment of H3K27ac at SOX2 intronic regions in sphere culture which was statistically significant for SOX2 intron 1 region 2 in both BER-DSRCT and JN-DSRCT-1. For CSF1 and TJP1, we found greater enrichment of H3K27ac in adherent culture, with TJP1 promoter region 2 showing a statistically significant enrichment in BER-DSRCT. These findings align with the known function of H3K27ac as a marker that increases chromatin accessibility and subsequently gene expression. They demonstrate that epigenetic alterations are involved in the transition from the adherent to the sphere state, including specific alterations in H3K27ac.

### BLK and LCK are enriched in the DSRCT CSC model and upregulated by EWSR1-WT1

**Fig. 5:**
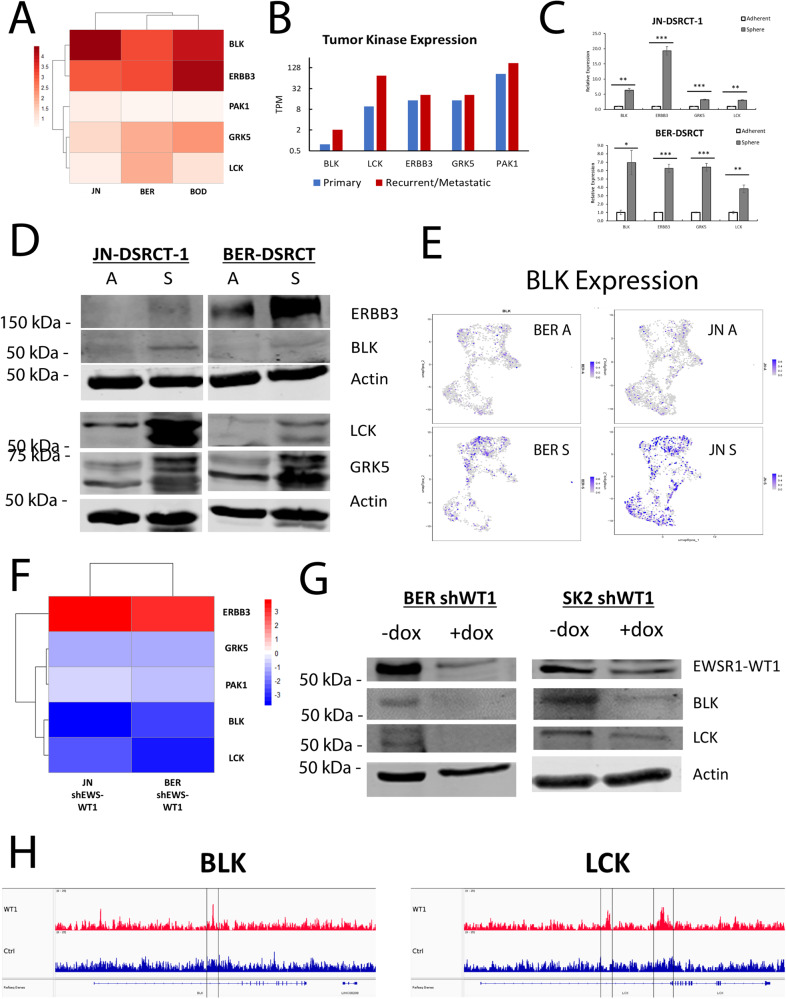
BLK and LCK are DSRCT CSC-enriched kinases. **A** Heatmap of log2FC (sphere/adherent) RNA expression of kinases upregulated in three DSRCT cell lines. **B** RNA expression of sphere-enriched kinases in primary and recurrent DSRCT tumors (TPM, *n* = 1). **C** Relative expression of sphere-enriched kinase RNAs in sphere versus adherent culture for JN-DSRCT and BER-DSRCT assessed via RT-qPCR (*n* = 3, *p < 0.05, **p < 0.01, ***p < 0.001, student *t* test). **D** Western blot of sphere-enriched kinase protein expression in the sphere and adherent culture conditions. **E** UMAP Projection of BLK expression in scRNA-seq data from BER- and JN-DSRCT-1 cells in (A) adherent or (S) sphere culture conditions. **F** Heatmap of sphere-enriched kinase gene expression change following depletion of *EWSR1-WT1* with siRNA (siEWS-WT1/ctrl). **G** Western blot of BLK, LCK, EWSR1**-**WT1, and ACTIN in BER-DSRCT shWT1 and SK-DSRCT2 shWT1 cell lines with or without dox to deplete EWSR1-WT1. **H** Previous ChIP-Seq data of EWSR1-WT1 binding sites in *BLK* and *LCK* in JN-DSRCT-1 cells.

We next sought to utilize our gene expression data to identify DSRCT CSC therapeutic targets. We focused our effort on kinases because they are frequently druggable and involved in important oncogenic processes, including proliferation, apoptosis, cell motility, and differentiation [30, 31]. We were particularly interested in kinases upregulated by EWSR1-WT1 as we previously demonstrated the importance of the *EWSR1-WT1* gene fusion to the CSC phenotype [8], and our ATAC-seq data found enrichment of the WT1 motif in altered accessibility peaks. Utilizing the sphere versus adherent RNA-seq data set, we identified 5 kinases as significantly upregulated in spheres versus adherent cells with >1.5 fold-change in all three cell lines: B lymphoid tyrosine kinase (BLK), Erb-B3 receptor tyrosine kinase (ERBB3/HER3), G-protein coupled receptor kinase 5 (GRK5), lymphocyte-specific protein tyrosine kinase (LCK), and p21 activated kinase 1 (PAK1) (Fig. 5A). To test the clinical relevance of these targets, TPM values for these kinases were compared using RNA-seq data from the pair of primary and recurrent tumors [9]. All five kinases were expressed at higher levels in recurrent than primary tumor samples, suggesting these targets may be clinically relevant and further validating the ability of the in vitro DSRCT CSC model to recapitulate clinically relevant gene expression alterations (Fig. 5B). RT-qPCR and western blot analyses confirmed the upregulation of BLK, ERBB3, GRK5, and LCK in sphere versus adherent culture at the transcriptional and translational level (Fig. 5C, D). Similarly, scRNA-seq showed higher expression of these four kinases in sphere versus adherent cells, with the highest BLK expression observed in sphere-enriched clusters 1 and 7 (Fig. 5E, Supplementary Fig. 5A–C). We examined ATAC-seq tracks of these 4 genomic locations to determine if increased expression of these genes is associated with increased chromatin accessibility. We found two SAPs in intron 1 of *GRK5* and an AAP in the *LCK* promoter (Supplementary Fig. 6A–D). No significantly altered peaks were found in *BLK* or *ERBB3*.

Using publicly available RNA-seq data of the siRNA knockdown of *EWSR1-WT1* in JN-DSRCT-1 and BER-DSRCT [6] (GSE137561), we discovered that *EWSR1-WT1* knockdown leads to prominent downregulation of *BLK* and *LCK*, modest downregulation of *GRK* and *PAK1*, and prominent upregulation of *ERBB3* (Fig. 5F). Since *EWSR1-WT1* is important for maintaining the CSC phenotype in DSRCT, the prominent downregulation of *BLK* and *LCK* might suggest that one or both kinases play a role as a downstream effector of *EWSR1-WT1* critical to the CSC state. To verify that *BLK* and *LCK* are positively regulated by *EWSR1-WT1* in spheres, our previously established doxycycline (dox)-inducible *EWSR1-WT1* knockdown cell lines were utilized: BER-DSRCT-shWT1 and SK-DSRCT2-shWT1 [8, 27]. These cells enable selective knockdown of the *EWSR1-WT1* fusion gene only when dox is added. Four days of treatment with dox led to the knockdown of *EWSR1-WT1* as well as *BLK* and *LCK* in both cell lines as compared to untreated controls (-dox) (Fig. 5G). Examining previously published ChIP-Seq data of EWSR1-WT1 binding sites in JN-DSRCT-1 cells [32] (GSE156277), we found that EWSR1-WT1 binds at one site in intron 1 of *BLK* and two sites in intron 1 of *LCK*, suggesting the fusion protein directly regulates transcription of these kinases (Fig. 5H).

To further explore the expression of *BLK* and *LCK* in DSRCT, we used publicly available microarray data from 137 patients with fusion-positive sarcomas [33], including DSRCT (*n* = 28), alveolar rhabdomyosarcoma (ARMS; *n* = 23), alveolar soft part sarcoma (ASPS; *n* = 12), Ewing sarcoma (ES; *n* = 28), and synovial sarcoma (SS; *n* = 46). *BLK* expression was higher in DSRCT than ARMS (*p* < 0.0001) and SS (*p* < 0.05), but lower than ES (*p* < 0.001) (Supplementary Fig. 5D). LCK expression was significantly higher in DSRCT than in all four other fusion sarcomas (*p* < 0.0001 for all tumors) (Supplementary Fig. 5E). Taken together, our comprehensive transcriptomic analysis identified *BLK* and *LCK* as (1) upregulated in sphere versus adherent culture, (2) more highly expressed in recurrent versus primary DSRCT tumor samples, (3) positively regulated by the *EWSR1-WT1* fusion gene, and (4) highly expressed in DSRCT as compared to other tumor types.

### SRC family kinase inhibitors reduce CSC characteristics

**Fig. 6:**
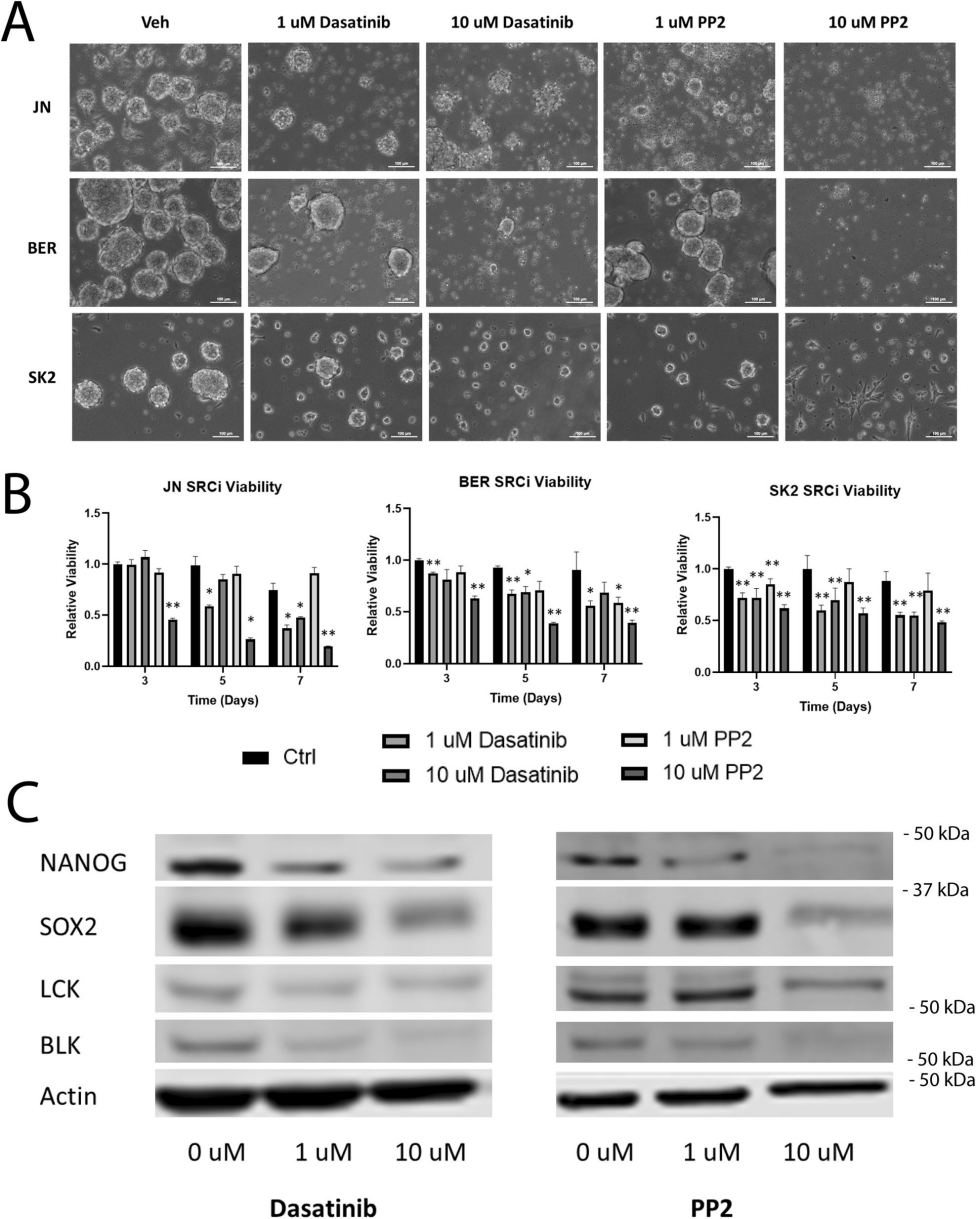
SRC kinase inhibitors reduce CSC characteristics. **A** Light microscopy of JN-DSRCT-1, BER-DSRCT, and SK-DSRCT2 cells grown in sphere culture conditions and treated for 7 days with vehicle control, 1 μM dasatinib, 10 μM dasatinib, 1 μM PP2, or 10 μM PP2 (scale bar = 100 µm). **B** Viability of DSRCT cells in sphere culture treated with SRC inhibitors for 3-, 5-, or 7 days (*n* = 3, *p < 0.05, **p < 0.01, ANOVA). **C** Western blot analysis of BER-DSRCT sphere culture cells treated for 7 days with vehicle control, 1 μM dasatinib, 10 μM dasatinib, 1 μM PP2, or 10 μM PP2.

*BLK* and *LCK* are both members of the SRC family kinases (SFKs), which also include *SRC*, *YES1*, *FYN*, *FGR*, *LYN*, and *HCK* [34]. SFKs share a common structure with SH2 and SH3 domains, followed by a C-terminal kinase domain [34]. SFKs have been implicated in several cancer types and are involved in the regulation of cell proliferation, differentiation, and migration [34, 35]. Because of their common structure, SFK members are susceptible to inhibition by the same compounds, including dasatinib and PP2 [36, 37]. To test the role of *BLK* and *LCK* in the DSRCT CSC phenotype, three DSRCT cell lines (JN-DSRCT-1, BER-DSRCT-, and SK-DSRCT2) grown in sphere culture were treated for 72 hours with the vehicle, 1 μM, or 10 μM of dasatinib or PP2. Treatment with either SFK inhibitor led to reduced sphere formation in a dose-dependent manner (Fig. 6A). Treatment with 10 μM of PP2 led to near complete abrogation of sphere formation in all three cell lines. SFK inhibitor treatment also led to viability reductions at 3, 5, and 7 days in all cell lines (Fig. 6B). Western blot showed reduced protein expression of CSC markers *NANOG* and *SOX2* in a dose-dependent manner with the treatment of dasatinib and PP2 (Fig. 6C, Supplementary Fig. 7A, B). Inhibitor treatment also resulted in decreased expression of *BLK* and *LCK*, suggesting inactivation by these inhibitors may also affect the protein stability.

### BLK knockdown reduces CSC characteristics

**Fig. 7:**
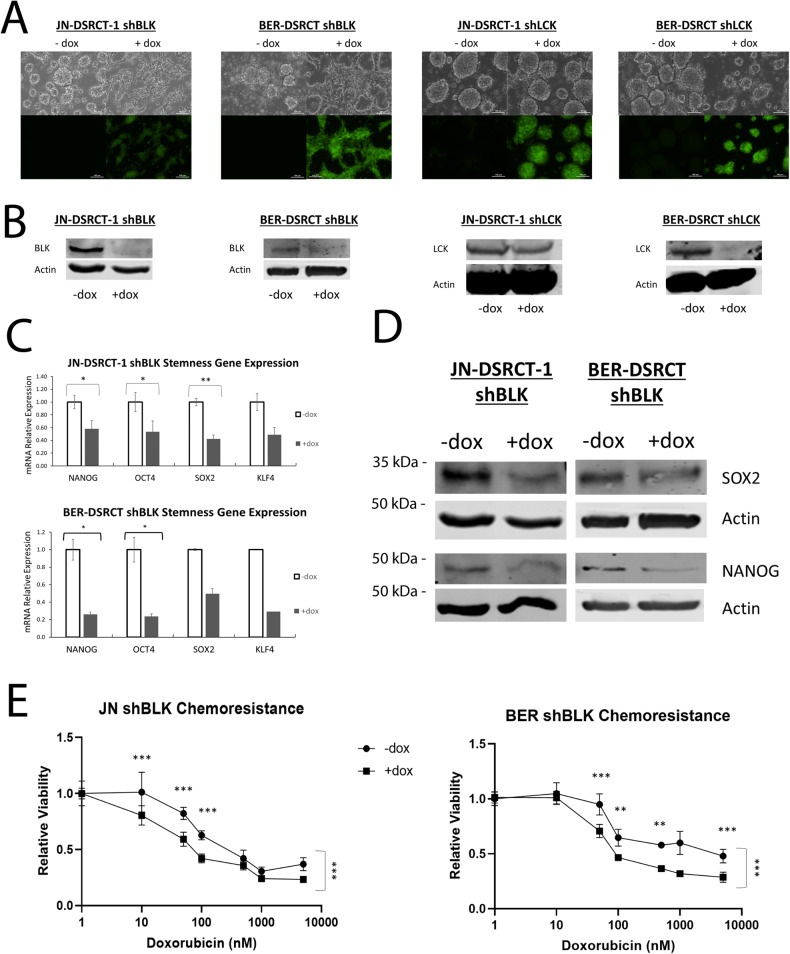
BLK knockdown reduces CSC characteristics. **A** Light and fluorescent microscopy of JN-DSRCT-1 and BER-DSRCT cells with dox-inducible shRNA to knockdown BLK or LCK. Cells were cultured in sphere conditions with or without dox for 7 days (scale bar = 100 µm). **B** Western blot of BLK and LCK expression in shBLK and shLCK cell lines with or without dox. **C** RT-qPCR analysis of *NANOG*, *OCT4*, *SOX2*, and *KLF4* transcripts in JN-DSRCT-1 and BER-DSRCT shBLK cell lines with or without dox (*n* = 3, *p < 0.05, **p < 0.01, ***p < 0.001, student *t* test). **D** Western blot of SOX2 and NANOG protein expression in shBLK cell lines with or without dox. **E** Relative viability of JN-DSRCT**-**1 and BER-DSRCT cell lines treated with doxorubicin for 72 hours with or without dox to silence BLK expression (*n* = 3, *p < 0.05, **p < 0.01, ***p < 0.001, ANOVA).

To examine the role of *BLK* and *LCK* in reduced stemness marker expression caused by SRC family kinase inhibitors, dox-inducible shRNA cell lines were established in JN-DSRCT-1 and BER-DSRCT (Fig. 7A). These cell lines selectively express green fluorescent protein (GFP) and shRNA when dox is added. Western blot and RT-qPCR confirmed the knockdown of *BLK* and *LCK* in the inducible shRNA cell lines with the addition of dox (Fig. 7B, Supplementary Fig. 8A). Intriguingly, depleting *BLK*, but not *LCK*, prevented sphere formation in JN-DSRCT-1 and BER-DSRCT (Fig. 7A). While shLCK cells formed spheres with or without the addition of dox, shBLK cells treated with dox adhered to the non-treated plate and displayed more of an adherent cell than sphere morphology. shBLK cells without dox readily formed spheres as expected. Consistent with the reduced ability to form spheres, *BLK* knockdown led to reductions in *SOX2*, *NANOG*, *OCT4*, and *KLF4* expression at the RNA level (Fig. 7C) and in *SOX2* and *NANOG* at the protein level (Fig. 7D). In contrast, silencing *LCK* did not lead to consistent reductions in stemness marker expression (Supplementary Fig. 8B, C). LCK depletion in JN-DSRCT-1 cells led to decreased expression of *NANOG* and *OCT4* at the transcriptional level, while in BER-DSRCT, *LCK* knockdown led to increased expression of *NANOG* and *OCT4* (Supplementary Fig. 8B). In both JN-DSRCT-1 and BER-DSRCT, *LCK* knockdown led to increased *SOX2* protein expression (Supplementary Fig. 8C). Together, these findings suggest that *BLK*, but not *LCK*, is the SFK sphere-enriched kinase that contributes to stemness properties in the DSRCT CSC model.

Previous work has demonstrated that DSRCT cells grown under CSC conditions have increased chemoresistance, especially to doxorubicin [8]. Having shown that silencing *BLK* decreases tumorsphere formation and stemness marker expression, we hypothesized that *BLK* knockdown could sensitize DSRCT cells to chemotherapy treatment. JN-DSRCT-1 and BER-DSRCT shBLK cell lines were grown in sphere culture with or without dox and treated for 72 hours with vehicle or doxorubicin doses from 10 nM to 10 μM. Depletion of BLK increased the sensitivity of JN-DSRCT-1 and BER-DSRCT cells to doxorubicin treatment (Fig. 6E). Similarly, treatment with 1 and 10 μM of dasatinib or PP2 further reduced cell viability caused by doxorubicin treatment (Supplementary Fig. 9). The role of *BLK* in both stemness marker expression and chemoresistance suggests *BLK* may play an important role in DSRCT recurrence and patient survival, making *BLK* an actionable target for DSRCT CSCs.

## Discussion

DSRCT is a highly aggressive tumor with high rates of recurrence and metastasis and poor patient prognosis. CSCs have the ability to resist chemotherapy, and targeting this population has the potential to overcome DSRCT’s ability to evade multimodal treatment. We recently established the first DSRCT CSC model and in this study, we utilized this model to gain insights into this subpopulation and identify potential DSRCT CSC therapeutic targets. RNA-seq analysis identified genes commonly upregulated and downregulated in the DSRCT CSC model. Remarkably, the genes altered in the DSRCT CSC model showed high concordance with genes altered in the only publicly available pair of RNA-seq data from primary and recurrent DSRCT tumors from the same patient. In fact, 8/18 upregulated pathways and 27/53 downregulated pathways in DSRCT CSCs were similarly altered in recurrent versus primary DSRCT tumors. This high concordance suggests the DSRCT CSC model is clinically relevant and could serve as an important tool for modeling recurrent DSRCT. Given the paucity of available data, more sets of primary versus recurrent DSRCT tumors are needed to further validate the DSRCT CSC model and provide greater insight into DSRCT recurrence.

Our multi-faceted transcriptomic analysis identified BLK and LCK as kinases that are enriched in the DSRCT CSC model, expressed highly in recurrent DSRCT, and regulated by the EWSR1-WT1 fusion protein. Depleting BLK reduced tumorsphere formation, decreased stemness marker expression, and sensitized the DSRCT CSC model to doxorubicin chemotherapy. Recently, Van Erp et al. treated JN-DSRCT-1 xenograft tumors with dasatinib in order to examine the effect of SRC kinase inhibition on DSRCT tumor growth [38]. The study failed to show a significant reduction in tumor volume with dasatinib treatment, suggesting inhibition of SRC as well as other SFKs, including BLK and LCK alone, is insufficient to kill DSRCT bulk tumors. This suggests *BLK* targeting therapy would be best used in combination with another therapy (e.g., chemotherapy) targeting bulk tumor cells. In this regard, it would be interesting to evaluate a combined therapy with dasatinib and recently identified DSRCT therapeutic targets NTRK3 [39] or SIK1 [27], which target the bulk tumor cells. The establishment of an in vivo DSRCT chemoresistance model would greatly advance DSRCT research and enable the examination of DSRCT CSC targets, including BLK.

In DSRCT, BLK has been previously identified by Gedminas et al. as 1 of 68 genes commonly upregulated by the *EWSR1-WT1* in JN-DSRCT-1 and BER-DSRCT cell lines [6]. Further, ChIP-seq from Hingorani et al. identified intron 1 of *BLK* as a direct binding location of the EWSR1-WT1 fusion protein [32]. In this study, we confirmed that BLK is regulated by EWSR1-WT1 by demonstrating reduced expression of BLK upon EWSR1-WT1 depletion. We further utilized both inhibitors and shRNAs to evaluate the functional role of BLK in DSRCT. Beyond DSRCT, BLK has been suggested as an oncogene in B and T-cell lymphomas [35, 40–42]. Malek et al. found that expression of constitutively active BLK in B and T cells of transgenic mice leads to the generation of B lymphoid tumors and thymic lymphomas [40]. Similarly, Petersen et al. demonstrated that ectopic expression of constitutively active BLK in the pro-B cell line Ba/F3 was sufficient to induce tumor formation in NOD/SCID mice, and that subsequent treatment with the SFK inhibitor dasatinib was able to significantly reduce tumor volume [35]. In line with these findings, dasatinib treatment dephosphorylated BLK in cutaneous T-cell lymphoma and reduced tumor growth in vivo [41]. However, to our knowledge, this is the first study establishing a role for BLK in solid tumors and demonstrating BLK’s effect on stemness properties, including tumorsphere formation, *SOX2* expression, and chemoresistance. The mechanism by which BLK modulates the CSC state remains unknown and warrants further investigation. Our finding that BLK knockdown reduces SOX2 mRNA levels, suggests a transcriptional mechanism, possibly through a yet-to-be-discovered intermediate that BLK phosphorylates. Phosphorylation of SOX2 may also play a role in maintaining the CSC state as SOX2 phosphorylation at T118 by AKT1 has been shown to prevent SOX2 degradation and promote embryonic stem cell reprograming [43]. BLK is known to phosphorylate CD79A, for which AKT1 is a downstream target [44]. This represents just one of the many potential mechanisms by which BLK may modulate SOX2 expression and the DSRCT CSC state.

BLK is a particularly interesting cancer target because its inhibition or knockdown is likely to have few side effects. BLK expression is largely confined to B cells where it is involved in B cell activation and proliferation [40, 45, 46]. Intriguingly, a study by Texido et al. showed that BLK is dispensable for these B cell-specific functions [47]. Utilizing a *Blk* knockout mouse, Texido et al. found that B cell development, in vitro activation, and humoral immune response all remained intact in the absence of BLK expression [47]. The authors posited that the function of BLK is redundant in B cells and may be accomplished by other SRC kinases in *Blk*-null mice. While it is possible that the role of BLK differs between mice and humans, the finding that BLK is dispensable for B cell development and activation in mice suggests that a specific BLK inhibitor, if developed, has the potential to target DSRCT CSCs with minimal adverse effects on humoral immunity.

Because of the low incidence of DSRCT, estimated at fewer than 50 cases per year in the United States, the development of DSRCT-specific therapeutics poses a significant financial barrier and is unlikely to be prioritized by pharmaceutical companies [48]. Therefore, repurposing existing medications is a promising path toward improving DSRCT treatment. While a *BLK*-specific inhibitor is lacking, inhibitors targeting BLK (along with other SFKs) exist and have already been tested in clinical trials. Dasatinib, utilized to inhibit BLK both in this study and in previous works, has been approved by the FDA for the treatment of chronic myelogenous leukemia and Philadelphia chromosome-positive acute lymphoblastic leukemia [36, 49–51]. Dasatinib is also under investigation for use in several solid tumors, including breast cancer, colorectal cancer, and prostate cancer [36]. Intriguingly, dasatinib has shown the ability to decrease the CSC population of breast cancer and colorectal cancer [52, 53]. While this effect has been attributed to its dephosphorylation of SRC, these studies have not verified the mechanism with a specific SRC knockdown, leaving open the possibility that the CSC reductions in these tumors may be due to SRC or some other SRC family kinase inhibited by dasatinib. Given our findings showing BLK inhibition sensitizes DSRCT CSC to chemotherapy and the existence of dasatinib as a drug previously FDA approved to target BLK, combination therapy with dasatinib and conventional chemotherapy or multiple targeted therapy is a promising DSRCT therapeutic regimen that warrants further investigation. BLK inhibition will target the CSC population, while chemotherapy eliminates bulk tumor cells, creating a two-pronged therapeutic strategy that accounts for DSRCT heterogeneity and can hopefully improve patient survival.

### Supplementary information

Supplementary Figures and Materials

Supplementary Data 1

Supplementary Data 2

## Data Availability

The RNA-seq, ATAC-seq, and scRNA-Seq datasets generated and analyzed during the current study have been made available on GEO at GSE248857.
