# Supplementary material for: Transcriptomic analysis identifies B-lymphocyte kinase as a therapeutic target for desmoplastic small round cell tumor cancer stem cell-like cells
Source: Oncogenesis. 2024 Jan 4;13(1):2. doi: 10.1038/s41389-023-00504-z (PMC10767073; doi:10.1038/s41389-023-00504-z)
Supplement: Supplementary file 1 — Supplementary Figures and Materials [file 41389_2023_504_MOESM1_ESM.docx]

**Supplementary Figures:**

**Supplementary Fig 1. DSRCT CSC pathway enrichment.** **(A)** Heatmap of sphere versus adherent RNA expression (log2FC) of stemness markers SOX2 and NANOG in JN-DSRCT-1, BER-DSRCT, and BOD-DSRCT **(B)** GSEA of GO-BP pathways in sphere versus adherent RNA-seq data.


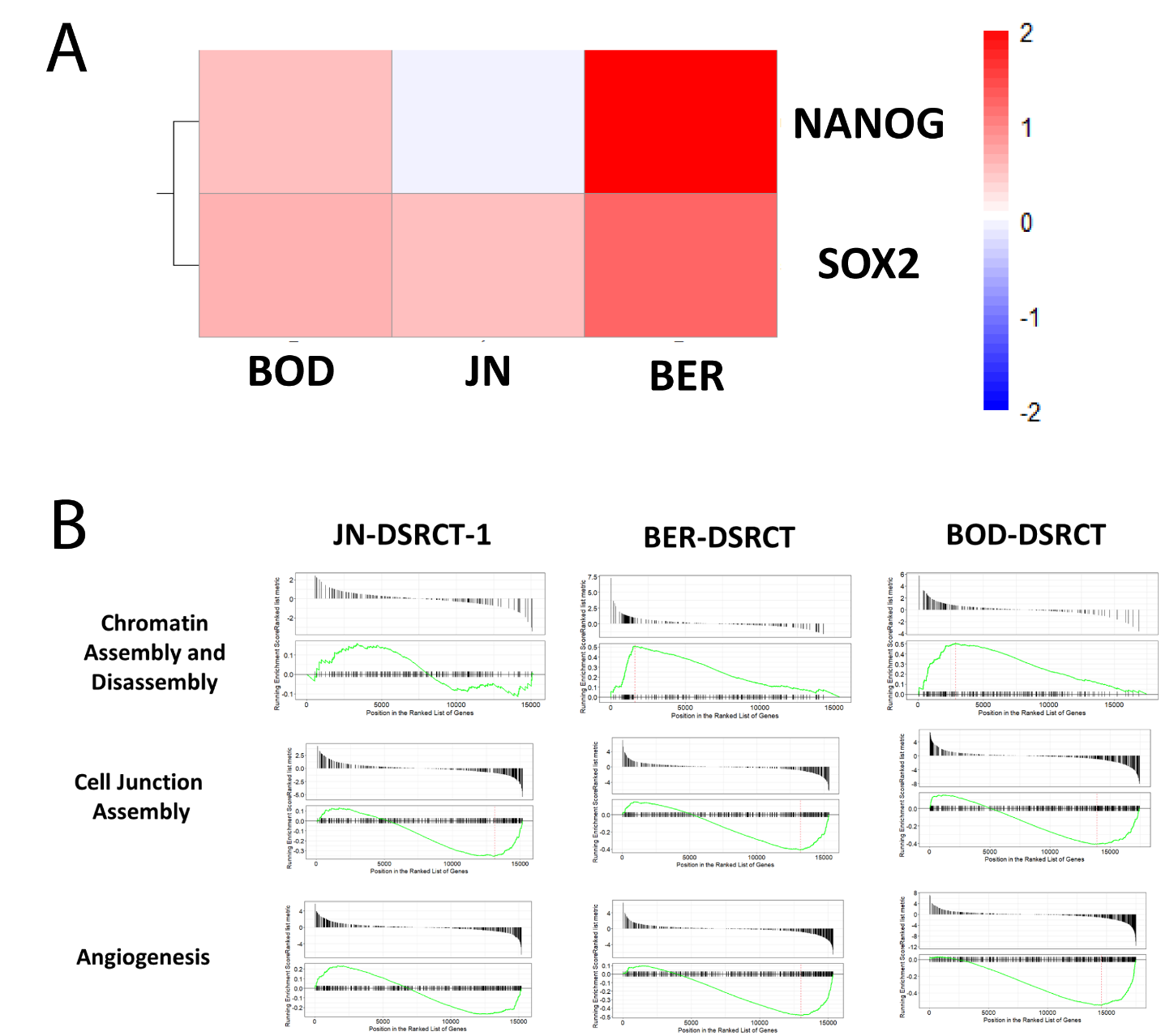


**Supplementary Fig 2. EWSR1-WT1 signature alterations.** **(A)** Heatmap of RNA expression changes (log2FC) of the EWSR1-WT1 regulated gene set in cell lines depleted of EWSR1-WT1 with siRNA (JN siWT1, BER siWT1) or cultured in sphere versus adherent conditions (BOD S v. A, BER S v. A, JN S v. A). **(B)** GSEA of EWSR1-WT1 upregulated and downregulated genes sets in cell lines cultured in sphere versus adherent conditions.


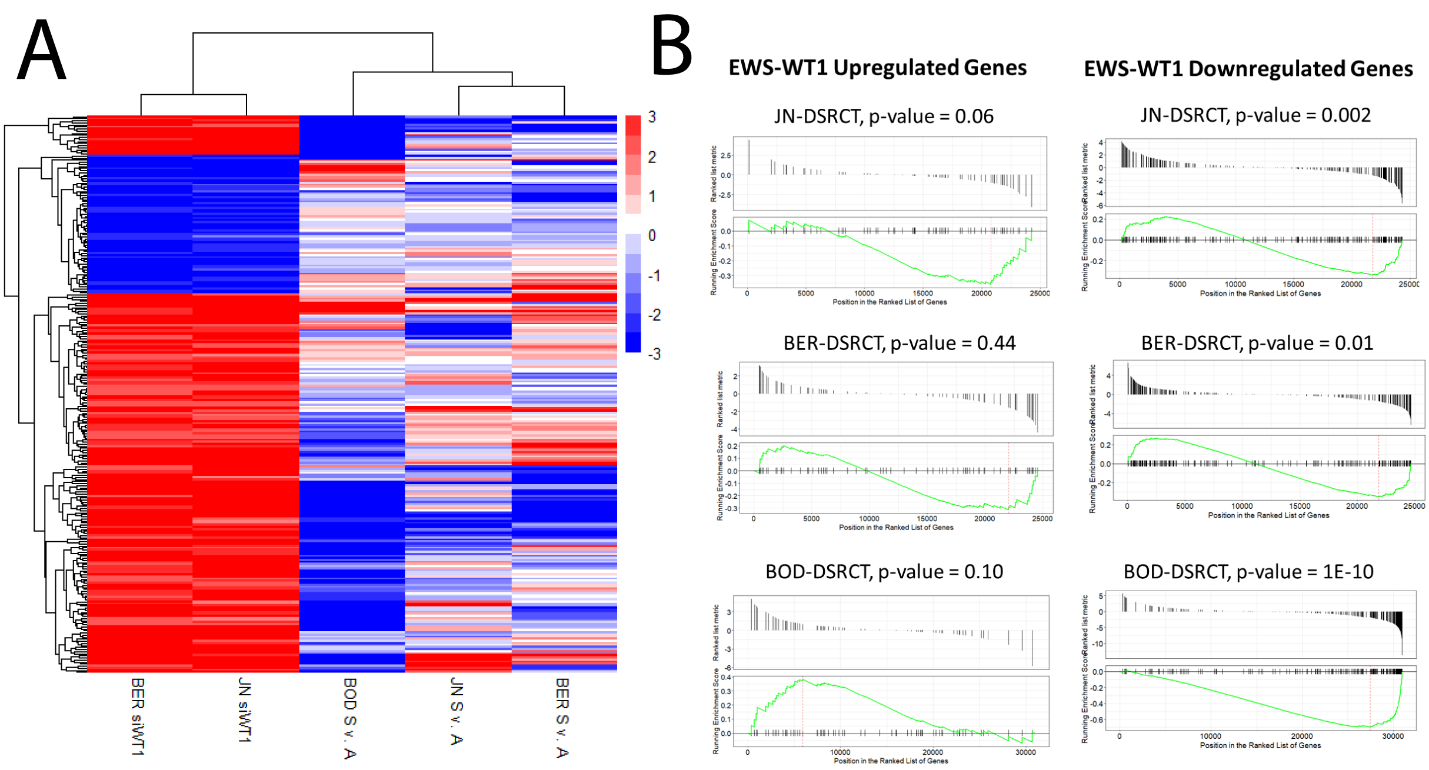


**Supplementary Fig 3. Single Cell Analysis.** **(A)** UMAP Projection of single cells from BER-DSRCT and JN-DSRCT-1 grown in sphere (S) or adherent (A) culture. **(B)** UMAP Projection showing SOX2 expression in single cells from BER-DSRCT and JN-DSRCT-1 grown in sphere (S) or adherent (A) culture.


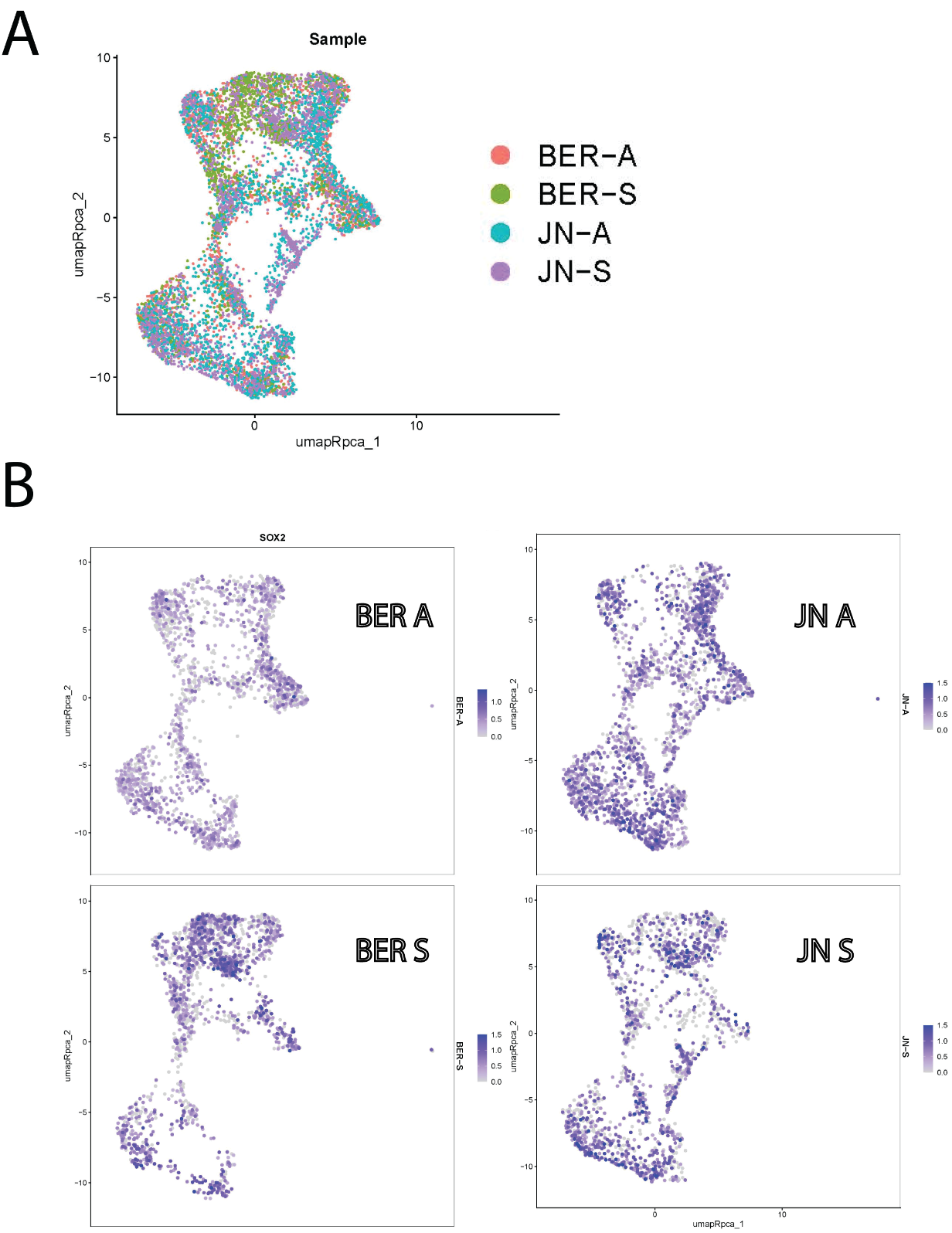


**Supplementary Fig 4. Motif Enrichment.** HOMER known motif enrichment of **(A)** sphere accessible peaks (SAPs) and **(B)** adherent accessible peaks (AAPs) relative to all peaks.


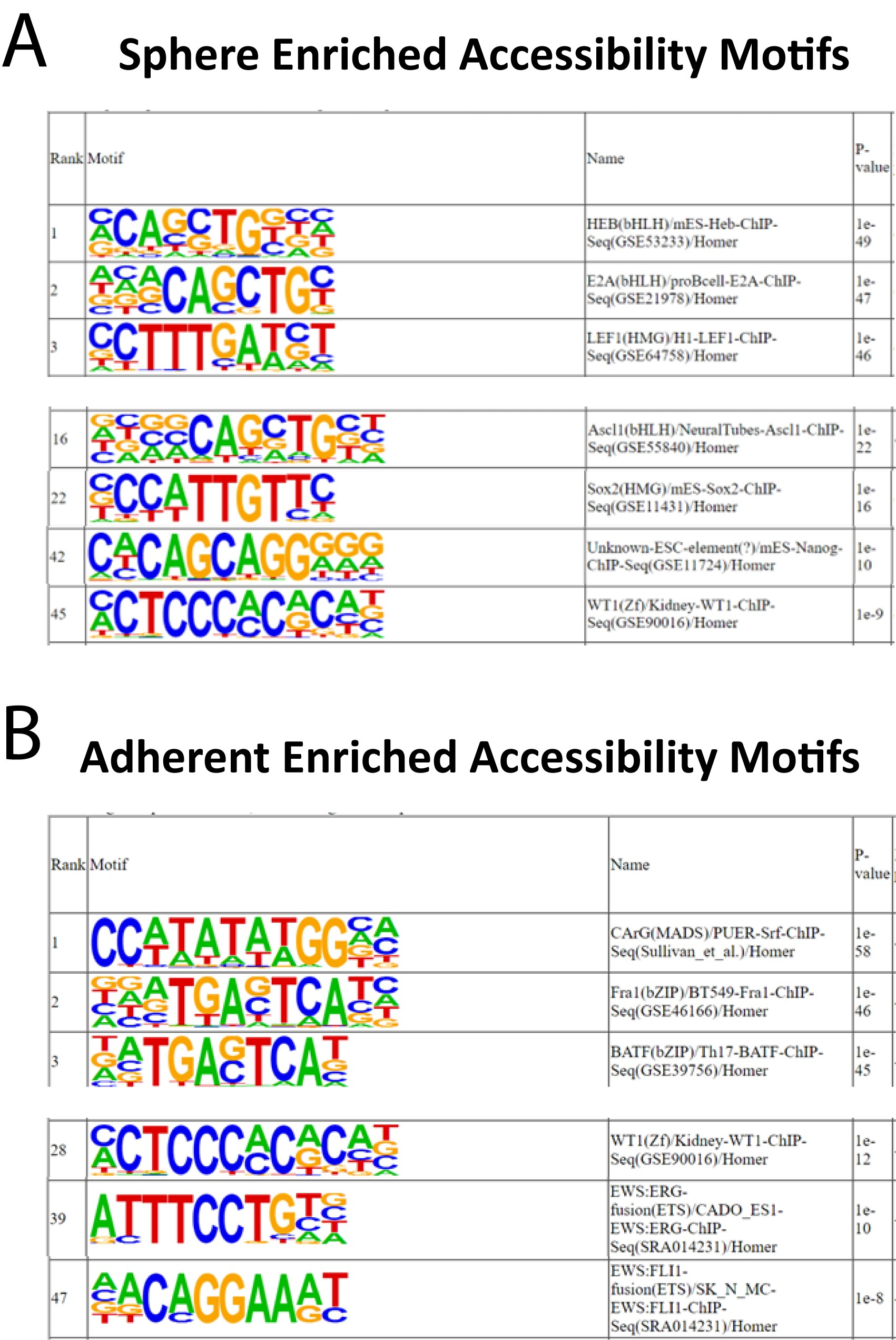


**Supplementary Fig 5. Sphere-enriched kinase expression.** **(A-C)** UMAP projections showing expression of LCK **(A)**, ERBB3 **(B)**, and GRK5 **(C)** in single cells from BER-DSRCT or JN-DSRCT-1 in sphere (S) or adherent (A) culture. **(D-E)** Relative transcript levels of **(D)** *BLK* and **(E)** *LCK* in DSRCT (n=28) ARMS (n=23), ASPS (n=12), ES (n=28), and SS (n=46) primary tumors based on Affymetrix U133A expression array data.


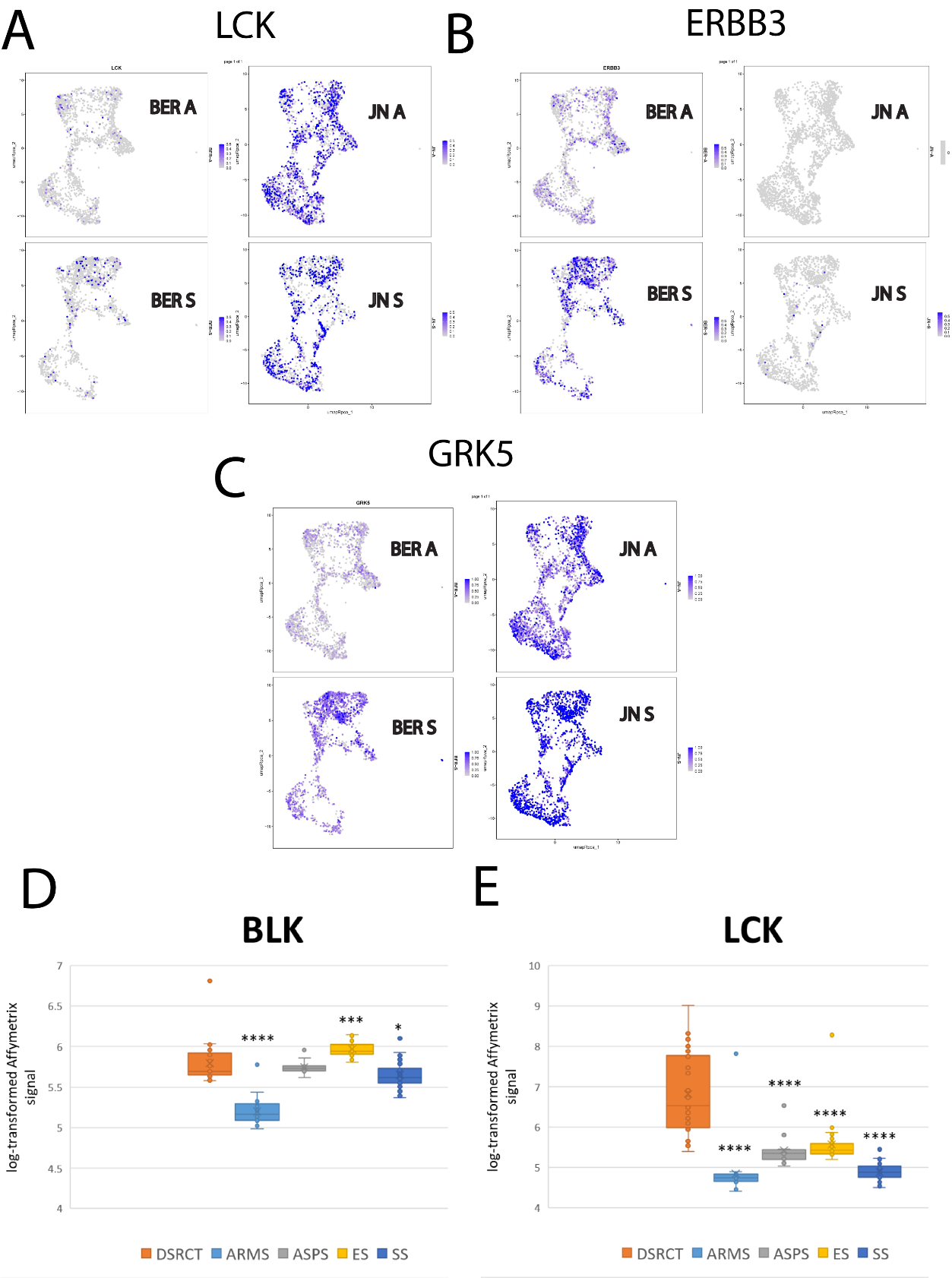


**Supplementary Fig 6. Sphere-enriched kinase accessibility.** **(A-D)** BER-DSRCT adherent (blue) and sphere (red) ATAC-seq tracks for **(A)** *BLK,* **(B)** *LCK,* **(C)** *GRK5,* and **(D)** *ERBB3.* Differentially accessible peaks are indicated by black bars.


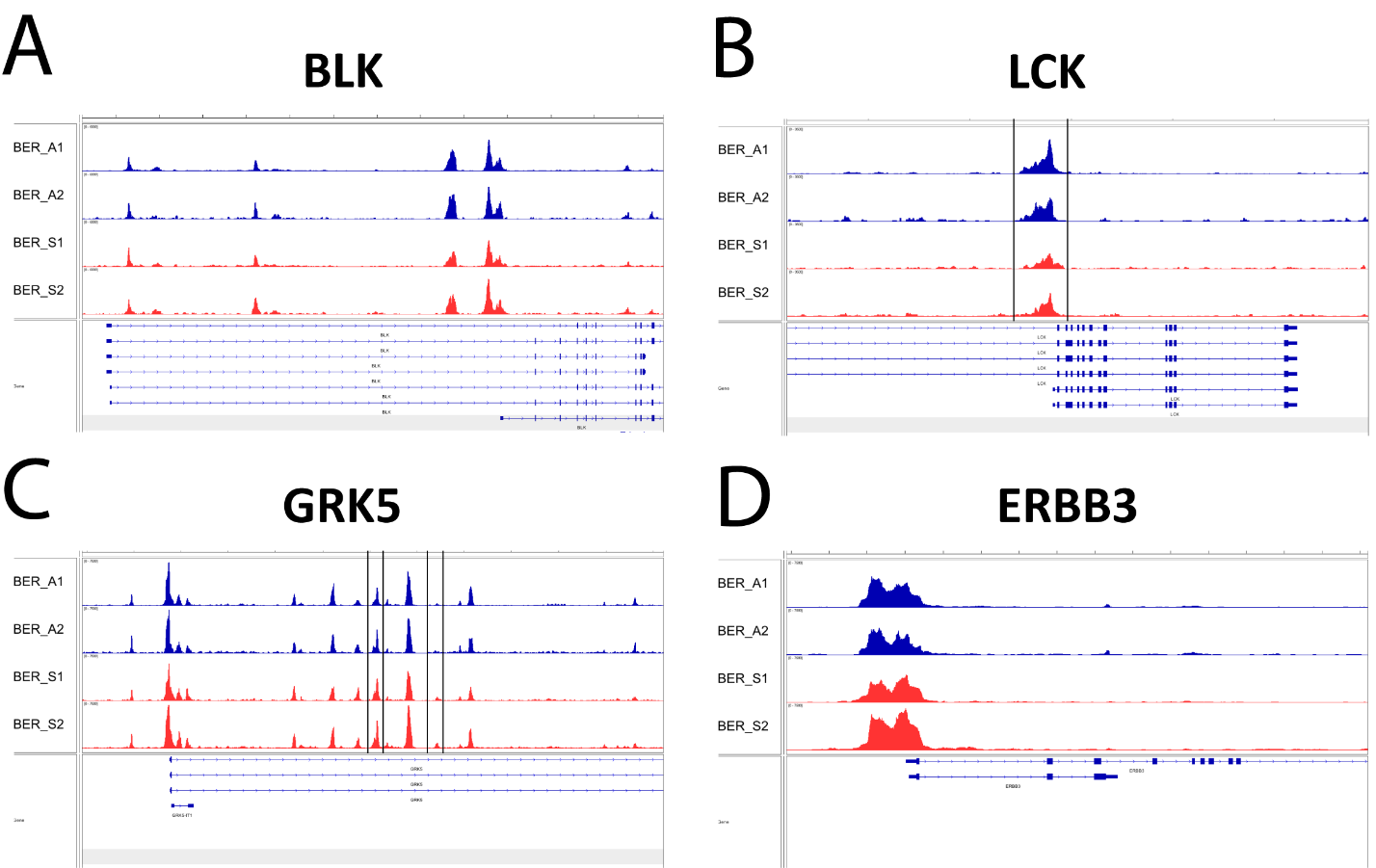


**Supplementary Fig 7. SRCi effect on stemness marker expression.** **(A-B)** Western blot analysis of **(A)** JN-DSRCT-1 and **(B)** SK-DSRCT2 sphere culture cells treated for 7-days with vehicle control, 1 μM dasatinib, 10 μM dasatinib, 1 μM PP2, or 10 μM PP2.


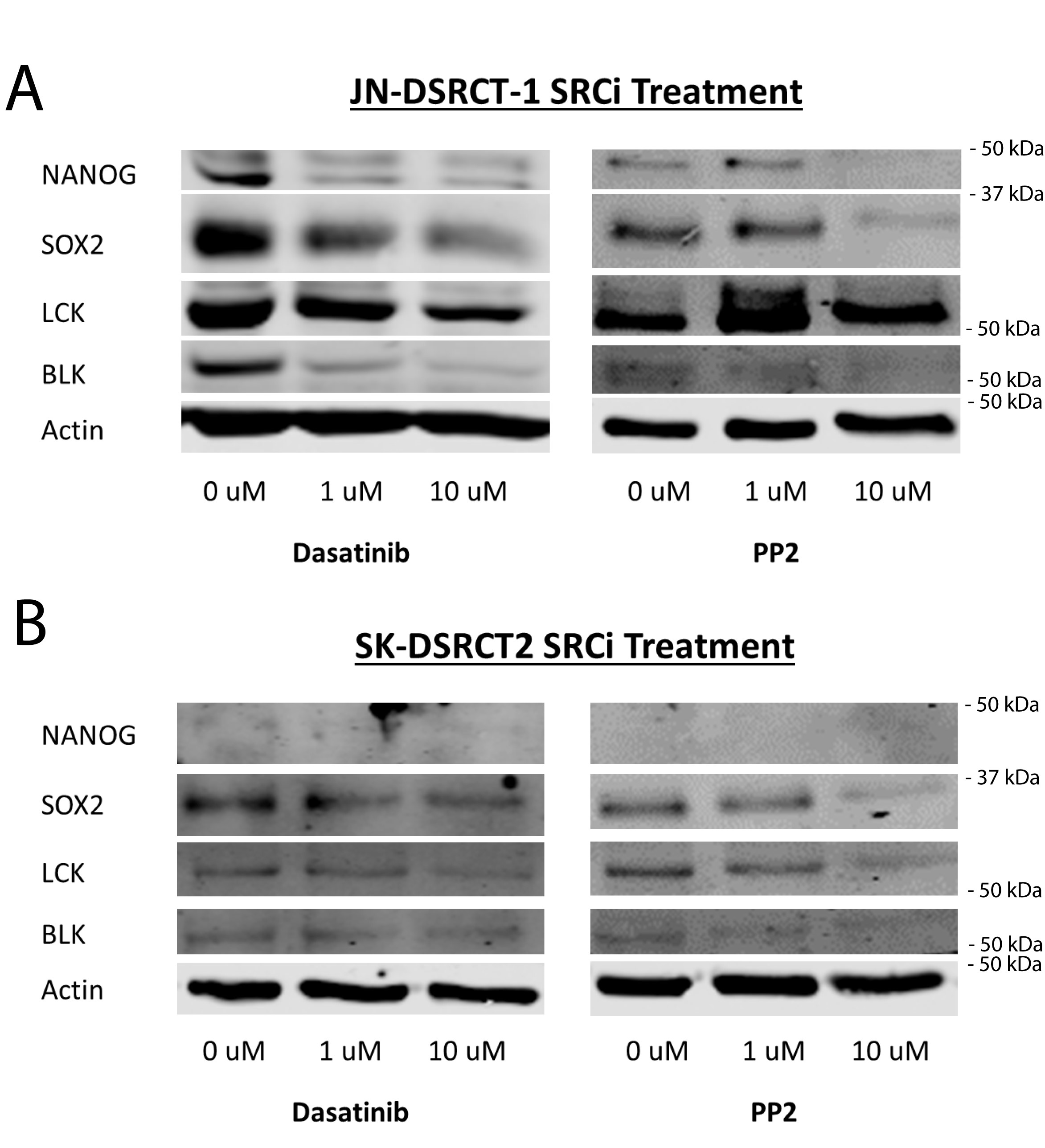


**Supplementary Fig 8. LCK knockdown does not alter stemness expression.** **(A)** RT-qPCR analysis of *BLK* or *LCK* transcripts in JN-DSRCT-1 shBLK, BER-DSRCT shBLK, JN-DSRCT-1 shLCK, and BER-DSRCT shLCK cell lines treated with or without dox (n=3, * p<0.05, ** p<0.01, *** p<0.001, student t-test). **(B)** RT-qPCR analysis of *NANOG*, *OCT4*, *SOX2*, and *KLF4* transcripts in JN-DSRCT-1 and BER-DSRCT shLCK cell lines with or without dox (n=3, * p<0.05, ** p<0.01, *** p<0.001, student t-test). **(D)** Western blot of SOX2 protein expression in shLCK cell lines with or without dox.


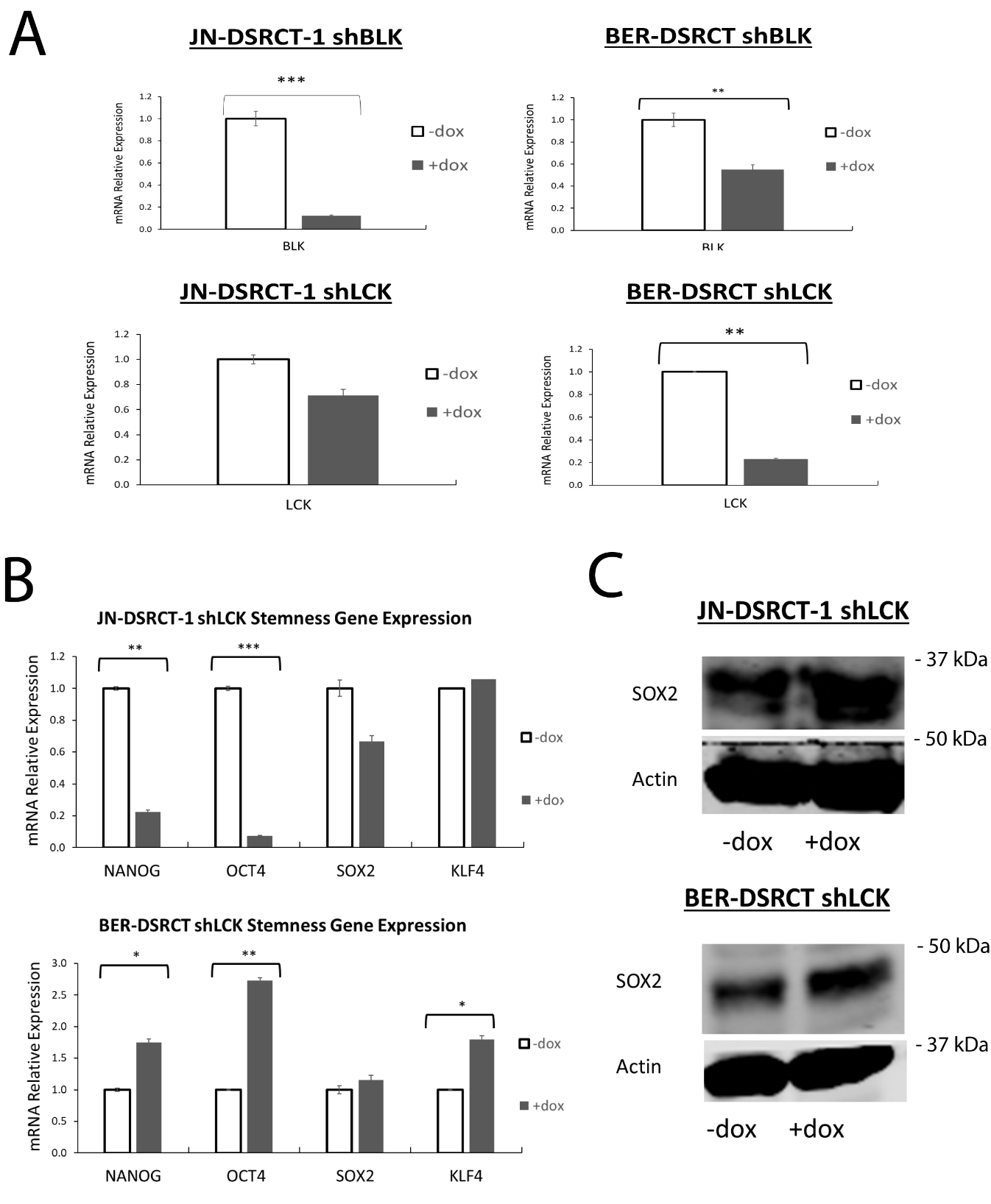


**Supplementary Fig 9. Combination therapy treatment in DSRCT CSCs.** **(A)** Relative viability of JN-DSRCT-1 and BER-DSRCT cells treated for 72-hours with a combination of dasatinib (0, 1, or 10 μM) and doxorubicin (10 nM to 10 μM). **(B)** Relative viability of JN-DSRCT-1 and BER-DSRCT cells treated for 72-hours with a combination of PP2 (0, 1, or 10 μM) and doxorubicin (10 nM to 10 10 μM).


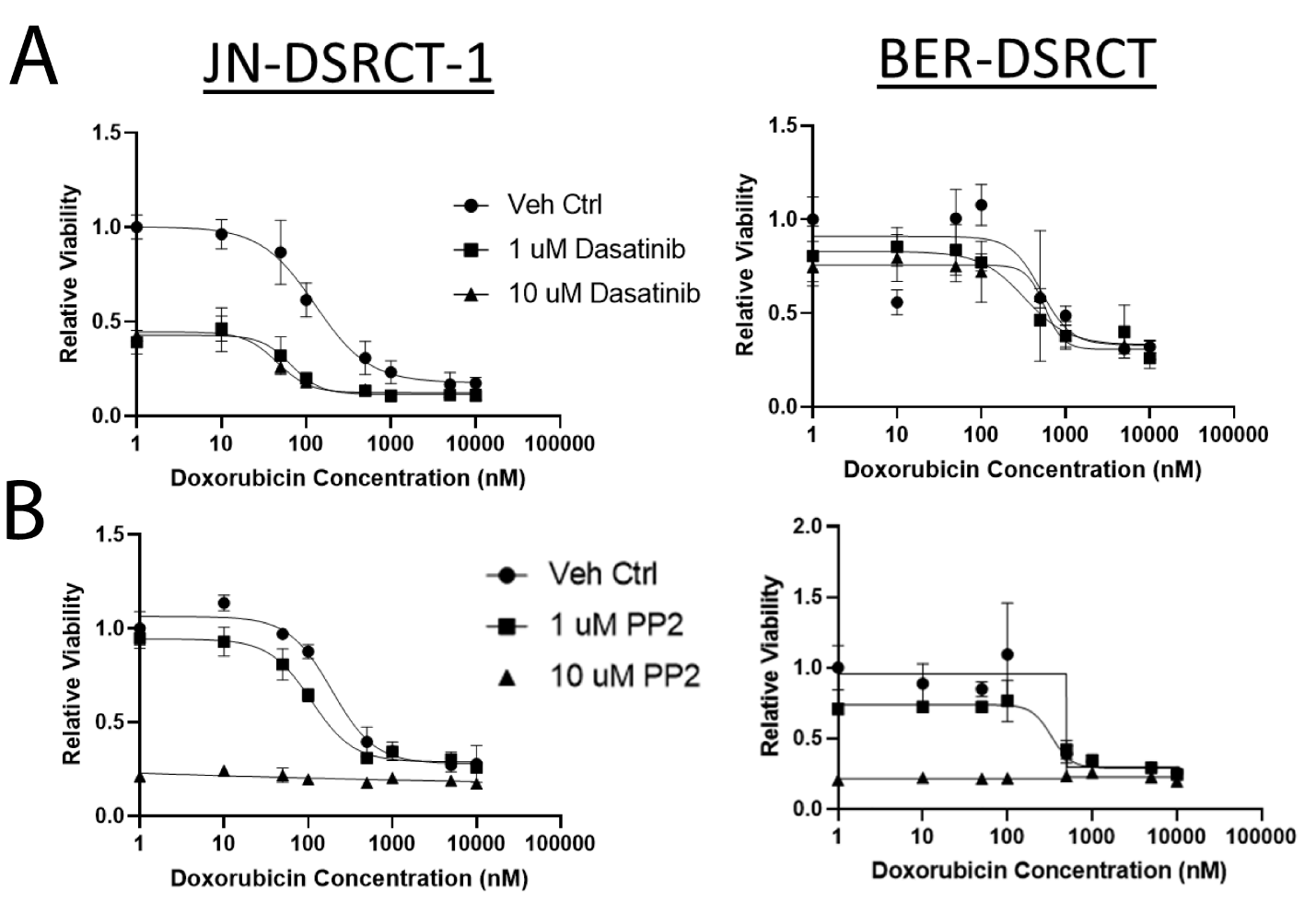


**Supplementary Tables**

**Supplementary Table 1:** RT-qPCR Primers

| **Gene** | **Fwd Primer (5’ -> 3’)** | **Rev Primer (5’ -> 3’)** |
| --- | --- | --- |
| ACTB | GCAAAGACCTGTACGCCAAC | AGTACTTGCGCTCAGGAGGA |
| NANOG | AGTCCCAAAGGCAAACAACCCACTTC | TGCTGGAGGCTGAGGTATTTCTGTCTC |
| POU5F1/OCT4 | GACAGGGGGAGGGGAGGAGCTAGG | CTTCCCTCCAACCAGTTGCCCCAAAC |
| SOX2 | GGGAAATGGGAGGGGTGCAAAAGAGG | TTGCGTGAGTGTGGATGGGATTGGTG |
| KLF4 | ACGATCGTGGCCCCGGAAAAGGACC | CAACAACCGAAAATGCACCAGCCCCAG |
| BLK | GGATGGGGCTGGTAAGTAGC | CTGCAGGTCCCGATCATTCA |
| LCK | GCTGACGGAAATTGTCACCC | TCAAGGCTGAGGCTGGTACT |
| ERBB3 | CAGCTCACCGAGATTCTGTC | GAGGAGCACAGATGGTCTTG |
| GRK5 | AGACCTCCGAAGGACCATAG | TCCTTCCCTTTCTCTCCCAG |

**Supplementary Table 2:** Antibodies for Western Blot and ChIP

| **Antibody** | **Company** | **Catalog #** | **WB Dilution** |
| --- | --- | --- | --- |
| β-Actin (8H10D10) | Cell Signaling | 3700 | 1:1000 |
| SOX2 (D6D9) | Cell Signaling | 3579 | 1:1000 |
| NANOG (D73G4) | Cell Signaling | 4903 | 1:500 |
| BLK | Cell Signaling | 3262 | 1:500 |
| HER3/ErbB3 (D22C5) | Cell Signaling | 12708 | 1:500 |
| LCK | Cell Signaling | 2657 | 1:1000 |
| GRK5 | Invitrogen | PA5-96262 | 1:1000 |
| H3K27Ac | Cell Signaling | 8173 | ChIP: (1:75) |

**Supplementary Table 3:** ChIP-qPCR Primers

| **Region** | **Fwd Primer (5’ -> 3’)** | **Rev Primer (5’ -> 3’)** |
| --- | --- | --- |
| SOX2 Intergenic Region 1 | CGTTACCAAGGCCTACCCTG | GGACGCTGATGGAGAGTCTG |
| SOX2 Intergenic Region 2 | GCCTTACTGTTGTGACTGCG | GCCACTAACTGTTCTCGTGG |
| SOX13 Intron 1 Region 1 | CAGGATAAACAAGGCCGCAC | CCTGAGACACCTGTTCTCGC |
| SOX13 Intron 1 Region 2 | CAGTGGGTGTGAGTGAGGTG | GGGGACAAAAGGAAGCCAGA |
| TFP1 Promoter Region 1 | GCTGGGTGGTCTCCTGATTC | CTGCGCTTCTTGTCCATGTG |
| TFP1 Promoter Region 2 | CACCAAGAGGCTGGGACATT | AGAGACTGCTGACCTGCCTA |
| CSF1 Intergenic Region 1 | ACACAGGGGAGGAAGGTTCT | CAGTGACCAGGCCTGATGTT |

**Supplementary Methods**

**scRNA-Seq Analysis:**

**1. scRNA-seq data processing**. Raw read processing was carried out using the Cell Ranger Single-Cell Software Suite (version 7.0.1, 10X Genomics Inc., CA, USA). The demultiplexed FASTQ files (paired-end, Read 1: 150bp, Read 2:150bp) were generated using the CellRanger mkfastq command. The primary data analyses which included alignment, filtering, barcode counting and UMI quantification for determining gene transcript counts per cell (generated a gene-barcode matrix), quality control, clustering and statistical analysis were performed using CellRanger count command.

2. **Single-cell gene expression quantification and filtering**. Raw gene expression matrices generated per sample using CellRanger were imported into R (version 4.2.2) and converted to a Seurat object using the Seurat R package (version 4.3.0). The ambient RNA was cleaned using DecountX (R package celda, Version 1.4.7). Dead cells and doublets were removed. The first, the total number of UMIs and genes, and percentage of UMIs derived from mitochondrial genome for each cell were counted. Then, Cells which had over 15% UMIs derived from mitochondrial genome were discarded. Next, the upper bound was calculated as mean plus two standard deviation (SD) and the lower bound as mean minus two SD for both the total UMIs and genes, respectively. Finally, Cells with total UMIs or genes outside of the upper and lower bounds were removed.

**3.Data integration (**rPCA) **and determination of the major cell types**. The remaining cells from 4 samples were integrated together and batch effects were correct using rPCA of Seurat . Firstly, for each sample, gene expression matrices were normalized to total cellular read count and Cell-Cycle scores were calculated using Seurat CellCycleScoring function. Then, Seurat SCTransform function was applied for the normalized data to remove cell cycle effect and select 2500 highly variably genes (HVG). Next common HVG for all samples are selected by function “*SelectIntegrationFeatures*”. After running “*PrepSCTIntegration*” and “*FindIntegrationAnchors*”, all data are integrated using “*IntegrateData*”. We checked HVG and removed mitochondria genes. Following that, we scaled and re-calculated PCA for cleaned HVG. The RunUMAP function was then applied to do the Uniform Manifold Approximation and Projection (UMAP) dimensional reduction. The FindNeighbors constructed a Shared Nearest Neighbor (SNN) Graph, and FindClusters function with “resolution = 0.8” parameter was carried out to cluster cells into different groups. The main cell types were identified on the basis of predicted and known marker genes acquired from the SingleR ([https://github.com/LTLA/SingleR) and](https://github.com/LTLA/SingleR)%20and) CellMarker database (http://biocc.hrbmu.edu.cn/CellMarker/).

4. **Identification of marker genes and differential expression genes (DEG).** To identify marker genes for these cell types, we compared the gene expression values of cells from the cluster of interest to that of cells from the rest of clusters using the Seurat FindMarkers function with default parameter of “MAST” test. Marker genes were defined based on the following criteria: 1) the average expression value in the cluster of interest was at least 1.2-fold higher than the average expression in the rest of clusters; 2) there are greater than 10% of cells in the cluster of interest which were detectable; and 3) marker genes should have the highest mean expression in the cluster of interest compared to the rest of clusters.

To calculate DEG between two group of cells, Seurat FindMarkers function with method “MAST” were applied for two group of cells with parameter “min.pct = 0.01, logfc.threshold = 0.01”. For marker genes and DEG lists, GO and pathway analyses were performed by R package ClusterProfile (V3.18.1)^16^.

5. **Gene set enrichment analysis**

Differentially expressed genes with adjusted p<0.05 were screened between clusters of cell types via “FindMarkers” function. “clusterProfiler” (https://doi.org/10.1016/j.xinn.2021.100141) was carried out to detect Gene Ontology (GO) and Kyoto Encyclopedia of Genes and Genomes (KEGG) pathways enriched by differentially expressed. The terms with false discovery rate (FDR) <0.05 were regarded as significant enrichment.
